# CircUbe3a from M2 macrophage-derived small extracellular vesicles mediates myocardial fibrosis after acute myocardial infarction

**DOI:** 10.7150/thno.52843

**Published:** 2021-04-15

**Authors:** Yan Wang, Chaofu Li, Ranzun Zhao, Zhimei Qiu, Changyin Shen, Zhenglong Wang, Weiwei Liu, Wei Zhang, Junbo Ge, Bei Shi

**Affiliations:** 1 Department of Cardiology, Affiliated Hospital of Zunyi Medical University, Zunyi 563000, China.; 2 Department of Cardiology, The Second Affiliated Hospital of Zunyi Medical University, Zunyi 563000, China.; 3 Department of Cardiology, Shanghai Institute of Cardiovascular Diseases, Zhongshan Hospital, Fudan University, Shanghai 200032, China.

**Keywords:** CircUbe3a, Small extracellular vesicles, miR-138-5p, Rhoc, Myocardial fibrosis.

## Abstract

**Objective:** This study aimed to explore the role of circular RNAs (circRNAs) in M2 macrophage (M2M)-derived small extracellular vesicles (SEVs) in myocardial fibrosis development.

**Methods**: The regulatory role of M2M-derived extracellular vesicles (EVs) was evaluated in a mouse model of acute myocardial infarction. Immunofluorescence, quantitative real-time PCR (RT-qPCR), nanoparticle tracking analysis, Western blot analysis and electron microscopy were used to identify macrophages, large extracellular vesicles (LEVs) and SEVs. The circRNA expression profiles of M0 macrophages (M0Ms) and M2Ms were determined by microarray analysis. Bioinformatic analysis, cell coculture and cell proliferation assays were performed to investigate the expression, function, and regulatory mechanisms of circUbe3a *in vitro*. qPCR, RNA immunoprecipitation (RIP), dual-luciferase reporter assays, RNA fluorescence *in situ* hybridization (RNA-FISH), Western blot analysis and a series of rescue experiments were used to verify the correlation among circUbe3a, miR-138-5p and RhoC.

**Results:** CircUbe3a from M2M-derived SEVs triggered functional changes in cardiac fibroblasts (CFs). CircUbe3a was synthesized and loaded into SEVs during increased M2M infiltration after myocardial infarction. The fusion of the released SEVs with the plasma membrane likely caused the release of circUbe3a into the cytosol of CFs. Silencing or overexpressing circUbe3a altered CF proliferation, migration, and phenotypic transformation *in vitro*. We confirmed that circUbe3a plays a crucial role in enhancing functional changes in CFs by sponging miR-138-5p and then translationally repressing RhoC *in vitro*. *In vivo*, the addition of M2M-derived SEVs or overexpression of circUbe3a significantly exacerbated myocardial fibrosis after acute myocardial infarction, and these effects were partially abolished by circUbe3a-specific shRNA.

**Conclusions:** Our findings suggest that M2M-derived circUbe3a-containing SEVs promote the proliferation, migration, and phenotypic transformation of CFs by directly targeting the miR-138-5p/RhoC axis, which may also exacerbate myocardial fibrosis after acute myocardial infarction.

## Introduction

Although timely and complete myocardial reperfusion is the most effective way to salvage the myocardium after acute myocardial infarction (AMI), myocardial fibrosis after AMI still results in adverse ventricular remodeling and a high mortality 1. Fibroblast proliferation and phenotypic transformation play crucial roles in myocardial fibrosis after AMI 2. During this process, myofibroblasts induce reparative fibrosis in the infarcted areas of the myocardium to support the structural integrity of the infarcted ventricle 3. Long-term repeated aseptic inflammation is considered an essential pathophysiological mechanism of myocardial fibrosis. After MI, macrophages are recruited to the heart and perform dual functions as key inflammatory components and central regulators of cardiac injury 4. Previous studies have revealed that the crosstalk between macrophages and cardiac fibroblasts (CFs) promotes cardiac remodeling 5. Infiltrated macrophages regulate the proliferation and activation of fibroblasts by secreting transforming growth factor-β (TGF-β) and interleukin-6 (IL-6) 6. Extracellular vesicles (EVs) are secreted by all kinds of cells, including macrophages, and may play an essential role in intercellular communication by transferring proteins, microRNAs (miRNAs), and long noncoding RNAs (lncRNAs) 7, 8. Circular RNAs (circRNAs) are also enriched and stable in exosomes (a type of EV) 9 and can be transferred into target cells 7, 10. However, the functions of circRNAs contained in macrophage-derived EVs in myocardial fibrosis have not been elucidated.

CircRNAs are implicated in the pathogenesis of various cardiovascular diseases and are considered intriguing targets for therapeutic intervention 11, 12. Emerging evidence implicates circRNAs in various physiological and pathological processes, such as cell survival, growth, differentiation, and metastasis. CircRNAs also regulate gene expression by acting as miRNA sponges, RNA-binding protein sequestering agents, or nuclear transcriptional regulators 13, 14. Unlike miRNAs and lncRNAs, circRNAs are structurally stable, are not easily degraded, and can function for a prolonged time. Therefore, we speculate that macrophages are involved in the process of left ventricular remodeling and that EV-associated circRNAs may play a leading role in this process.

The current study examined the roles of macrophages in cardiac fibrosis using a murine model of AMI. M2 macrophages (M2Ms) accumulated after 7 days of MI and promoted excessive fibrosis in cardiac tissue. We also investigated whether and how EVs derived from M2Ms function in myocardial fibrosis after AMI, focusing on the mediating role of circUbe3a.

## Materials and Methods

### Animal model of AMI

Mice were first anesthetized with 40 mg/kg of sodium pentobarbital (Sigma, USA) via intraperitoneal injection and were subjected to AMI by ligation of the left anterior descending coronary artery (LAD) as described previously 15, 16. Seven days after LAD ligation, the mice were injected intramyocardially with 1 µg/g of EVs or PBS (n = 10) in a total volume of 20 μL at 5 different sites in the peri-infarcted area 17. To maintain the concentration of EVs in the myocardium, mice receiving the indicated treatments were additionally injected with 200 μg of 1,1'-dioctadecyl-3,3,3',3-tetramethylindocarbocyanine perchlorate (DiI)-labeled EVs via the tail vein (TV) for 7 days after the operation 17. All the mice were followed up 4 weeks after MI induction to assess left ventricle (LV) functional changes via echocardiography and structural remodeling assessment.

All the mice were maintained at the Experimental Animal Center of Zunyi Medical University, Gui Zhou. The animal experiments were conducted according to the Guide for the Care and Use of Laboratory Animals published by the US National Institutes of Health (NIH publication no. 85-23, revised 1996) and were approved by the Animal Care and Use Committee of Zunyi Medical University. All the studies involving animals have been reported in accordance with the ARRIVE guidelines.

### Transmission electron microscopy (TEM)

AMI tissue was cut into small 1-mm^3^ pieces and then was fixed with electron microscopy fixation solution and 1% osmium tetroxide in the dark. After dehydration, the samples were placed in a pure embedding agent for 12 h, embedded in resin, and baked in an oven for 12 h at 37 °C. Subsequently, the samples were moved into a heated polymerization apparatus and polymerized at 65 °C for 48 h. The samples were cut into thin slices (60- to 100-nm thick). Tissue sections were dyed first with uranium acetate for 1 h and then with citrate for 20 min. Finally, TEM was used to visualize the microstructure.

### Isolation and polarization of macrophages

Bone marrow-derived macrophages were flushed out from the femurs and tibias of 6-week-old mice and differentiated in complete Dulbecco's modified Eagle's medium (DMEM)/F12 medium supplemented with 50 ng/mL of macrophage colony-stimulating factor (MCSF) 18. On day 7, the macrophages were treated with IL-4 for 24 h to generate M2 macrophages 19. TEM (Hitachi H7500; Tokyo, Japan) and flow cytometry (FCM) were performed to identify M2 polarization.

### CF culture

Primary cultures of mouse CFs were prepared as previously described 20. Briefly, the ventricles from mice were cut into pieces of approximately 1-mm^3^ using ophthalmic scissors and digested with 0.125% trypsin (Invitrogen). Dispersed cells were incubated in a 25-cm^2^ culture bottle for 30 min in a 5% CO_2_ incubator. CFs attached to the bottom of the dishes were subsequently incubated in DMEM supplemented with 10% fetal calf serum for an additional 2 to 4 days. Confluent cells were treated with pancreatic enzymes (containing 0.25% trypsin and 0.02% EDTA) and subcultured. Confluent CFs grown in culture dishes from passages 2 to 4 were > 99% positive for vimentin, as assessed by labeling with anti-vimentin antibodies (Sigma). Serum-containing medium from the cultured cells was replaced with serum-free medium for 24 h to synchronize the cell cycle for subsequent experiments.

### Cell coculture

To evaluate the effect of M2Ms on CF proliferation *in vitro*, a cell coculture model was established 21. M2Ms were incubated overnight in transwell chambers, which were then inserted into another 24-well culture plate containing CFs that had been cultured at a density of 2 × 10^4^ cells/well for 24 h. The two cell types in the transwell chambers were cocultured for 36 h for further experiments. To clarify whether EVs are involved in the M2M-induced functional changes in CFs, 10 µM GW4869 (Sigma, USA) was used to reduce EV release from M2Ms. Before M2Ms were cocultured with CFs, they were stimulated with GW4869 for 8 h.

### Isolation and internalization of EVs

The procedures for isolating EVs from M2Ms were performed as previously described 10. TEM and immunoblotting were used to identify EVs. FCM was used to detect the EV surface markers CD9 and CD63. Alix, HSP70, and TSG101 were used as protein markers. Nanoparticle tracking technology was used to determine the particle size and concentration. A bicinchoninic acid (BCA) protein assay kit (Pierce) was used to quantify the amount of EVs, and DiI (Sigma) was used to label and track EVs. The pelleted EVs were resuspended in PBS and cultured in CF culture medium for 24 h for subsequent experiments.

**Figure 1:**
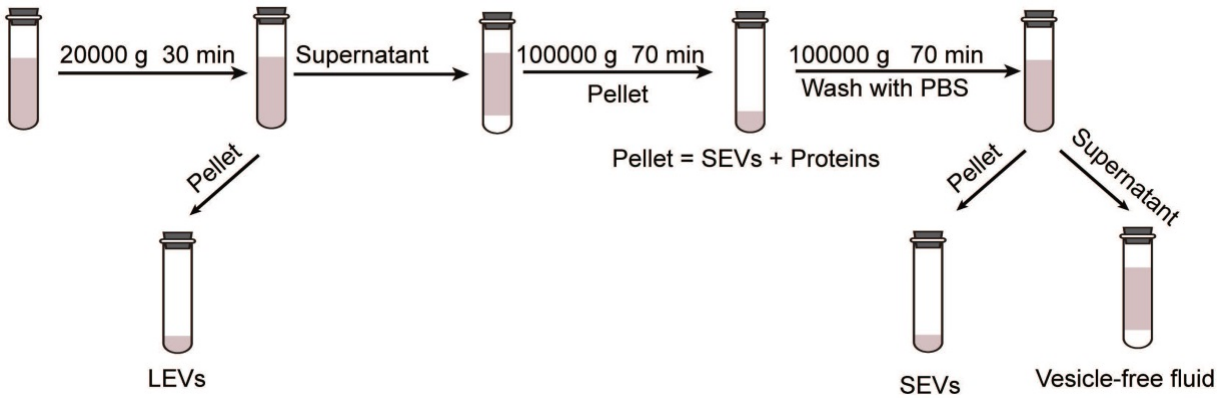
Schematic diagram of the procedure for EV extraction.

According to the International Society of Extracellular Vesicles (ISEV), EVs can be classified as large vesicles (> 200 nm) and small vesicles (< 100 nm or < 200 nm) rather than as exosomes and microvesicles 22. Large extracellular vesicles (LEVs) and small extracellular vesicles (SEVs) were extracted using different ultra-high-speed differential centrifugation methods (as shown in Figure 1). Nanoparticle tracking technology was used to determine the particle size and concentration. Subsequent studies were performed to further determine the effects of LEVs and SEVs on CFs.

### RNA extraction and microarray analysis

Total RNA was extracted from M0 macrophage (M0M)-derived SEVs (M0M-SEVs) and M2M-derived SEVs (M2M-SEVs) using TRIzol reagent (Life Technologies, USA), and then ribosomal RNA was removed using a ribosomal RNA removal kit (Thermo Fisher Scientific, USA). Ribonuclease (RNase) R working solution was mixed at a ratio of 4 U to 1 μg of RNA sample and incubated at 37 °C for 3 h to remove linear RNA. Digestion was terminated using a phenol-chloroform-isopropanol mixture, and the sample was then purified using an RNA purification kit. After preparing the RNA-seq library using Illumina's NEBNext Ultra Directional RNA Library Prep Kit according to the manufacturer's instructions, the differentially expressed circRNAs in M0M-SEVs and M2M-SEVs were analyzed using circRNA microarrays (Aksomics, China). Next, differential expression analysis of the microarray data was performed using the Limma R Studio Package, and differentially expressed genes were identified as those meeting the criteria of |logFC| >2 and *P*<0.05. Next, a corresponding heatmap was produced using the pheatmap package in RStudio.

### Detection of circUbe3a circularization

CircRNAs are more stable than linear RNAs because of their unique covalently closed circular structure and are not easily degraded by exonucleases or RNase R 23. Currently, experiments are being conducted primarily to verify the stability of the circular structure of circRNAs and sequence information of the loop at the backsplicing site to confirm the “circular” identity 24, 25. Because a circRNA is circularized from a precursor mRNA produced by its homologous parent gene, its sequence is homologous to that of the parent gene. However, the homologous linear mRNAs do not have a backsplicing site sequence similar to that of circRNAs during loop formation 24, 26; thus, circRNAs can be specifically amplified based on this characteristic. Because no circular sequences encoding circRNAs in genomic DNA (gDNA) and complementary DNA (cDNA) are contained in the transcriptome, linear and circular sequences can coexist 27, 28. Exploiting this difference, we designed forward and reverse primers specific for circUbe3a to separately amplify gDNA and cDNA and then performed gel electrophoresis. Two grams of total RNA was digested with 3 μ/g RNase R at 37 °C for 15 min and then was reverse transcribed into cDNA. The expression of circUbe3a after digestion was assessed by agarose gel electrophoresis, and a gel imaging system was used to scan and analyze electrophoretic bands under ultraviolet light.

### Cell transfection

The circUbe3a overexpression, circUbe3a siRNA, miR-138-5p inhibitor, and miR-138-5p mimic vectors, as well as the corresponding negative controls (NCs), were synthesized by GeneSeed (China). At 40%~60% confluence, cells (3×10^5^) were seeded in 6-well plates and transfected using Lipofectamine 2000 (Invitrogen, USA) or infected with lentiviruses according to the manufacturer's instructions. We also used different doses of GFP-labeled lentiviruses to obtain the optimal multiplicity of infection (MOI) in a preliminary experiment. Infected cells were refreshed with new culture medium for subsequent experiments.

### Prediction of circRNA-miRNA interactions

Interactions between circUbe3a and miR-138-5p were predicted using Arraystar's proprietary miRNA target prediction software, which is based on the miRanda and TargetScan databases. RNA fluorescence *in situ* hybridization (RNA-FISH) and dual-luciferase reporter assays were used to confirm both colocalization in the cytoplasm and binding sites.

### RNA-FISH

RNA-FISH was performed strictly according to the manufacturer's instructions 10, 29. After prehybridization (1× PBS/0.5% Triton X-100), CFs were hybridized overnight with FITC- or Cy3-labeled RNA probes targeting circUbe3a or miR-138-5p in hybridization buffer (40% formamide, 10% dextran sulfate, 1× Denhardt's solution, 4× saline-sodium citrate (SSC), 10 mM dichlorodiphenyltrichloroethane, 1 mg/mL yeast transfer RNA, and 1 mg/mL sheared salmon sperm DNA). Cell nuclei were counterstained with DAPI, and fluorescence images were acquired.

### Dual-luciferase reporter assays

For reporter assay analyses, 2×10^4^ cells (HEK293T) in a 96-well plate were transfected with 50 nM miR-138-5p or the mimic NC (RiboBio). The cells were then cotransfected with 2 µg/mL vector expressing the wild-type or mutant CDKN1B or CDKN1C 3'-UTR. After 48 h, luciferase activity was measured using a Dual-Luciferase® Reporter Assay System (Promega, Madison, WI, USA) according to the manufacturer's instructions.

### Transwell assays

Briefly*,* 100 μL of the CF suspension (1×10^5^ cells/mL) was dispensed into the transwell inserts (8-μm pore size; Costar, USA), and 600 μL of complete medium (with or without M2-SEVs) was added to the lower chamber. The transwell plates were incubated at 37 °C in a 5% CO_2_ incubator for 24 h. Subsequently, nonpenetrating cells on the top surface of the transwell insert membrane were wiped off, and the migrated cells on the bottom surface of the membrane were fixed with 4% paraformaldehyde for 30 min and stained with 1% crystal violet for another 15 min. Five randomly selected fields on each membrane were imaged using an inverted microscope (magnification, 10×).

### EdU incorporation assay

The Cell-Light 5-Ethynyl-20-deoxyuridine (EdU) DNA Cell Proliferation Kit (RiboBio, China) was used to detect cell proliferation according to the manufacturer's protocol. After incubation with 50 mM EdU for 2 h, proliferating cells were stained with Apollo Dye Solution and then were mounted with a mounting medium containing Hoechst 33342. Next, the EdU-positive cells in five randomly selected fields were imaged and counted under an Olympus FSX100 microscope (Olympus, Tokyo, Japan).

### ELISA

After cell coculture for 24 h, the concentrations of collagen I and collagen III in the medium were determined using ELISA kits (R&D, USA). Detection was performed strictly according to the manufacturer's instructions.

### Quantitative real-time PCR (RT-qPCR)

The relative mRNA expression of circUbe3a, miR-138-5p, and RhoC was analyzed as previously described. Total RNA was extracted from cell lysates or SEVs using the TRIzol one-step method (Invitrogen, USA). The purity of the isolated RNA was determined by the optical density 260/280 ratio using a NanoDrop ND-2000 spectrophotometer (Thermo Scientific). The isolated RNA was reverse transcribed using a MiRNA qRT-PCR Starter Kit (RiboBio, China). RT-qPCR analyses were performed using SYBR Premix Ex Taq II (TaKaRa). RNase R treatment was used for circRNA detection as previously described. Stem-loop RT-qPCR TaqMan MicroRNA assays (Life Technologies) were used to determine the miRNA amounts. GAPDH or U6 was used as the internal reference. All the primer sequences are listed in Table 1. Gene expression was quantified using the 2^-∆∆Ct^ method.

### Echocardiography

After the mice were anesthetized with a mixture of 1.5% isoflurane and oxygen (1 L/min), a Vevo 770 imaging system (VisualSonics, Toronto, Canada) equipped with a 30 MHz transducer was used to evaluate mouse cardiac function. The ejection fraction (EF) and fractional shortening (FS) were measured in the short-axis view from M-mode recordings.

### Western blot analysis

Western blot analysis of total protein from cardiac endothelial cells was performed as previously described. The extracted proteins were separated by sodium dodecyl sulfate-polyacrylamide gel electrophoresis (SDS-PAGE) and transferred onto PVDF membranes. After blocking overnight in a nonfat milk solution, the membranes were probed first with primary antibodies specific for collagen I, collagen III, α-SMA, RhoC, β-actin, and GAPDH and then with horseradish peroxidase-conjugated secondary antibodies for 1 h. Next, the PVDF membranes were incubated with enhanced chemiluminescence reagent (Amersham Biosciences, USA) before detection using a ChemiDoc MP system.

### Histological studies

For histological analysis, the mice were euthanized by an overdose of Avertin (200 mg/kg). Hematoxylin and eosin (H&E) and Masson's trichrome staining were performed according to standard protocols. The hearts were fixed with 10% buffered formalin and then were embedded in paraffin before being examined 28 days after MI induction. Morphometric analysis was performed on tissue sections using ImageJ software. The fibrotic area was measured to determine the percentage of fibrosis 11.

### Immunofluorescence staining

Immunofluorescence staining of tissue sections was performed as described previously 30. Nuclei were counterstained with 4',6-diamidino-2-phenylindole (DAPI; Sigma Aldrich, St. Louis, MO), and 10 randomly selected fields in each sample were imaged by fluorescence microscopy (Olympus, Tokyo, Japan). Anti-cTnT and anti-vimentin antibodies were purchased from Abcam (Cambridge, MA), and fluorescent secondary antibodies were obtained from Servicebio (Wuhan, China).

### Statistical analysis

The data were processed using the SPSS 21.0 statistical package (IBM, Armonk, NY, USA). The measurement data were normally distributed, and the results were expressed as means ± standard deviation. Comparisons among multiple groups were performed using one-way analysis of variance (ANOVA) followed by the least significant difference (LSD) test or Dunnett's T3 post hoc test for multiple comparisons. Additionally, *P*<0.05 was considered to indicate a significant difference.

## Results

### 1. Effect of EVs released from M2Ms on myocardial fibrosis *in vivo*

**Figure 2:**
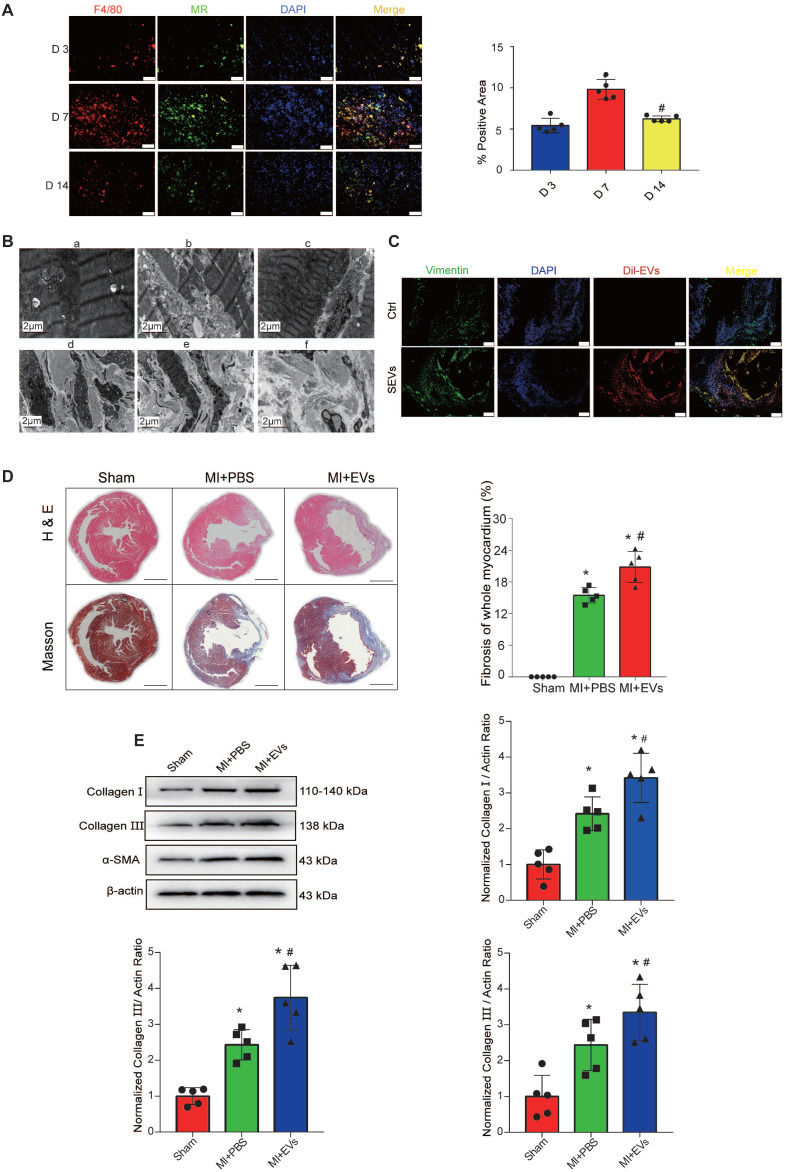
** M2M-derived EVs promote myocardial fibrosis. A:** Representative fluorescence micrographs of M2Ms in the infarcted area on D3, D7, and D14. Scale bar = 50 μm. **B:** Cardiac microstructure as revealed by TEM.** C:** DiI-labeled M2M-EVs were injected into mice via the intramyocardial and tail vein routes for 72 h. The relative fluorescence intensity (red) was quantified to assess the uptake of EVs. Scale bar=50 μm. **D:** H&E and Masson's trichrome staining assays were performed to visualize the morphology of myocardial tissues from MI rats. Scale bar = 200 μm. (*P < 0.05 versus sham; ^#^*P* < 0.05 versus MI + PBS; n=5 per group). **E:** Representative Western blots and quantification of the levels of collagen-related proteins (collagen I, collagen III, and α-SMA) in infarcted myocardial tissues treated with PBS or EVs (*P < 0.05 versus sham, ^#^*P* < 0.05 versus MI + PBS; n = 3 per group).

The number of M1 macrophages (M1Ms) gradually increases in the early stage of MI, peaks at day 3, and then gradually decreases. By contrast, the number of M2Ms peaks at day 7 of MI and continues to be detectable until day 28 31. Additionally, macrophages directly contribute to the postinjury fibrotic response by activating CFs to ensure that myofibroblast-derived collagen deposition is increased to advance and complete mature scar formation 31. Fibrosis is crucial for cardiac structural maintenance in the early stage of MI, but continuous fibrosis can lead to unfavorable ventricular remodeling. To explore the specific mechanism by which macrophages regulate cardiac fibrosis in the late stage of MI, we first verified the positions of macrophages and fibroblasts. As previously described 31, a few M2Ms were recruited on day 3 after AMI; the numbers peaked on day 7, and many M2Ms were still detectable on day 14 (Figure 2A). TEM showed that the myocardial cells in the sham-operated group were neatly arranged, the structure of the intracellular mitochondria was clear, and the sarcomere structure was obvious (Figure 2B-a). However, the myocardial arrangement was disordered and severely damaged, the myocardial fibers were broken, and the mitochondria began to swell in the infarcted area immediately after MI (Figure 2B-b). One week after MI, mitochondrial compensation was increased in the junction between the noninfarcted and infarcted regions (Figure 2B-c). Myofibroblasts were detected in cardiac tissues 7 days after AMI (Figure 2B-d) and secreted increased amounts of collagen I and collagen III (Figure 2B-e and 2B-f). These data verified a potential regulatory relationship between M2Ms and fibroblasts.

To verify that the role of M2M-mediated fibrosis after MI depends on EV release, DiI-labeled M2M-EVs were injected into mice for 7 days after the operation via the intramyocardial and tail vein routes, and the distribution of DiI-labeled EVs in the heart was then observed by immunofluorescence after 72 h. DiI-labeled EVs were successfully transferred to the heart and taken up by CFs (Figure 2C) and local cardiac cells (Figure S1). Subsequently, echocardiography was used to evaluate the effects of the different EV injection methods on cardiac function in mice. Compared with those of sham group mice, the EF and FS of AMI group mice were significantly decreased. Compared with those of AMI group mice, the EF and FS of mice receiving intramyocardial injection (MI) of M2M-derived EVs (M2M-EVs) were further reduced. Moreover, MI combined with the tail vein injection of M2M-EVs resulted in the lowest EF and FS values (Figure S2). The H&E and Masson's trichrome staining results showed that the myocardial fibrosis levels in the EV group were significantly increased compared with those in the PBS group (*P* < 0.05) (Figure 2D). Additionally, Western blot analysis was used to determine the expression levels of proteins associated with myocardial fibrosis. Compared with those in the sham group, the protein expression levels of collagen I, collagen III, and α-SMA were significantly increased in the PBS and EV groups (*P* < 0.05) (Figure 2E). Compared with those in the PBS group, the protein expression levels of collagen I, collagen III, and α-SMA in the EV group were further increased (*P* < 0.05). Thus, M2M-EVs participated in mediating myocardial fibrosis in the model of MI.

### 2. M2Ms contribute to functional changes in CFs by delivering EVs *in vitro*

To clarify whether M2Ms-EVs are involved in proliferation, migration, and phenotypic transformation in CFs, we cocultured M2Ms with CFs in a transwell system, which allowed the transfer of cellular secretions but prevented the transfer of vesicles larger than EVs and direct cell contact. GW4869 was used to reduce EV release from M2Ms. First, M0Ms obtained by primary culture of bone marrow were detected by F4/80 immunofluorescence (Figure S3A). Polarization of M0Ms to M1Ms was induced with LPS, and macrophage surfaces were labeled with iNOS, IL-1β, and TNF-α (Figure S3A, S3B). IL-4 successfully induced the polarization of M0Ms to M2Ms, and M2Ms expressed the surface markers MR and Arg (Figure S3A, S3B). Scanning electron microscopy showed that M0Ms appeared as flat rods with a small number of tentacles (Figure S3C), while M1Ms were slender and had long tentacle-like projections (Figure S3C). M2Ms were spherical and had fewer projections than M1Ms (Figure S3C).

**Figure 3:**
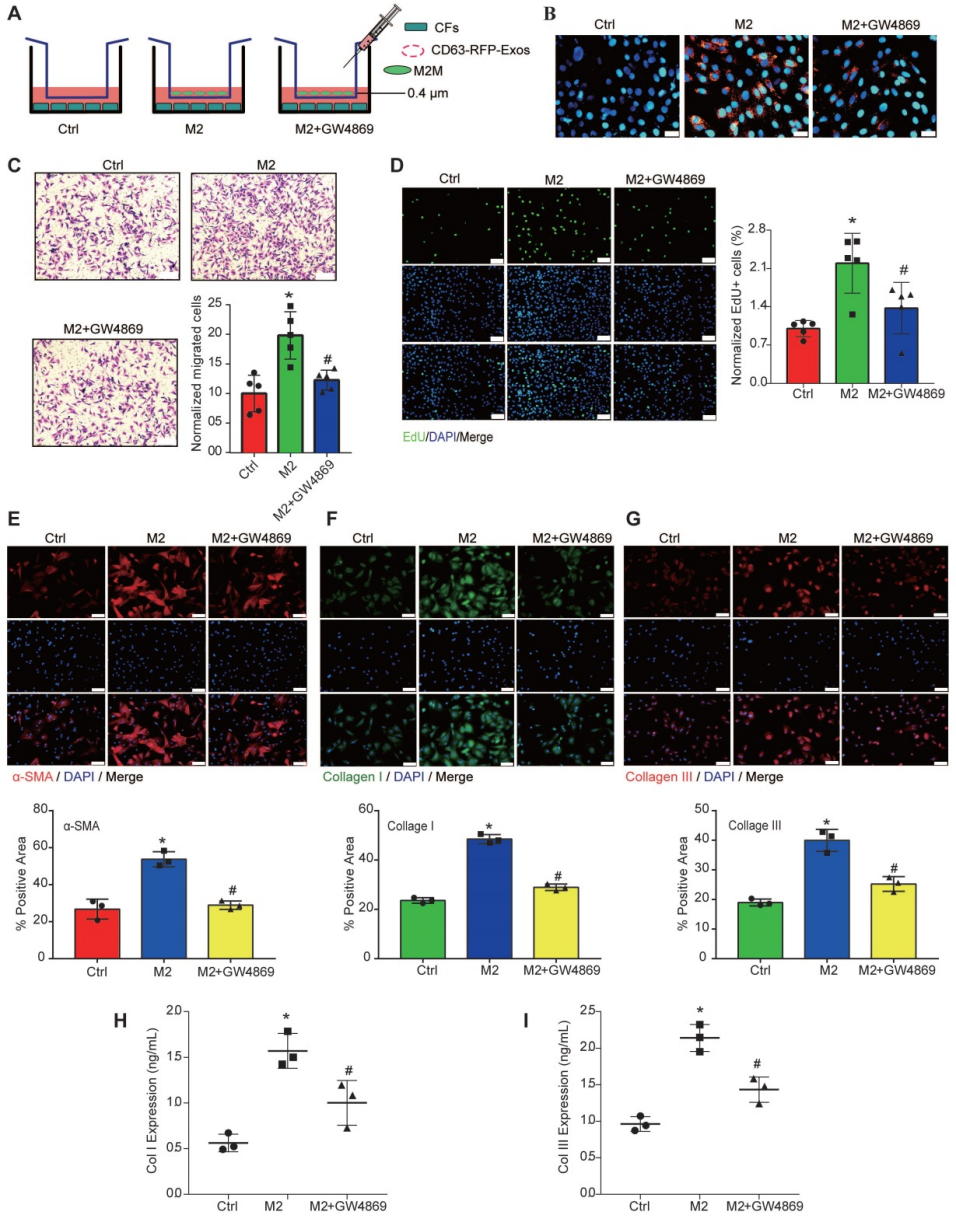
** CF function is affected by M2M-derived EVs.** GW4869, a well-known inhibitor of EV secretion, was used at 10 μM to reduce EV release from M2Ms. **A:** Schematic model of the coculture system used to show that M2Ms affect CF proliferation. **B:** RFP^+^ EVs were introduced into CFs. **C:** The transwell assay revealed a positive association between M2M-EVs and CF migration. Scale bar = 20 μm. **D:** The EdU assay showed a positive association between M2M-derived EVs and CF proliferation. Scale bar = 20 μm. **E:** Representative fluorescence micrographs of α-SMA in CFs and quantitative analysis after different treatments. Scale bar = 50 μm. **F:** Representative fluorescence micrographs of collagen I and quantitative analysis of CFs after different treatments. Scale bar = 50 μm. **G:** Representative fluorescence micrographs of collagen III in CFs and quantitative analysis after different treatments. Scale bar = 50 μm. **H:** The concentration of collagen I in the coculture system medium was measured by ELISA.** I:** The concentration of collagen III in the coculture system medium was measured by ELISA (*P < 0.05 versus the Ctrl group; ^#^*P* < 0.05 versus the M2 group; n = 3 per group).

Subsequently, we transfected M2Ms with EV-CD63-RFP tracer lentiviruses to label EVs with red fluorescent protein (RFP). After coculture with CFs for 48 h, large amounts of RFP^+^ proteins were observed in CFs via fluorescence microscopy, while the amount of RFP^+^ proteins in CFs was significantly reduced after GW4869 treatment, suggesting that GW4869 successfully blocked EV release (Figure 3A). The results of transwell, EdU, and immunofluorescence assays showed that CFs cocultured with M2Ms showed enhanced proliferation ability and expressed more collagen than those cultured alone. After adding GW4869 to inhibit the release of EVs, the abilities of CFs to proliferate, migrate, and express collagen in the coculture system were significantly reduced (Figure 3C-3I). These data suggested that M2Ms contribute to functional changes in CFs and that EVs may be involved in this process.

### 3. M2Ms contribute to functional changes in CFs by delivering SEVs *in vitro*

To verify the effect of EVs on fibroblasts of different sizes, ultra-high-speed differential centrifugation was used to obtain LEVs and SEVs. By TEM, LEVs were visualized as spherical vesicle-like vesicles with a diameter of approximately 200 nm (Figure S4A); SEVs were visualized as spherical saucer-shaped or hemispherical membraned structures with a diameter of approximately 30-120 nm (Figure S4A). No vesicle-like structures were observed in the supernatant (Figure S4A). Nanoparticle tracking analysis (NTA) of the size distribution characteristics of each M2M-EV component indicated an LEV particle size of 245.2±6.7 nm and an SEV particle size of 104.2±3.5 nm (Figure S4B). DiI (red)-labeled LEVs and SEVs were internalized by CFs (Figure S4C), and the EdU assay showed that LEVs and SEVs at the same particle concentrations promoted CF proliferation (*P*<0.05). SEVs induced a more significant increase in the proliferation of CFs than LEVs (Figure S4D). Additionally, Western blot analysis confirmed that SEVs had a stronger ability to promote the expression of α-SMA, collagen I, and collagen III in CFs (Figure S4E). Next, we explored the effects of SEVs derived from various macrophage types on the proliferation, migration, and phenotypic transformation of fibroblasts. The EdU and transwell assay showed that M2M-SEVs markedly promoted the proliferation and migration of CFs (Figure S5A, S5B). The above results indicated that M2M-SEVs had the greatest ability to regulate CF proliferation, migration, and phenotypic transformation.

Considering that M2Ms are polarized from M0Ms, we further explored the specific mechanism of M0M- and M2M-SEVs in the regulation of fibroblast proliferation, migration, and phenotypic transition. SEVs were purified from conditioned medium by ultracentrifugation of M0Ms and M2Ms. As shown in Figure S6A, all M0M- and M2M-SEVs exhibited a typical cup-shaped morphology following TEM analysis and ranged in size from 30 to150 nm. CD9 and CD63 are common specific markers of SEVs, and the percentage of isolated SEVs positive for these markers was determined using a direct fluorescent antibody assay combined with nanosensitive FCM. All the SEVs were positive after index staining, as determined by a threshold for positive staining greater than 70% (Figure S6B). Next, the expression of the SEV markers Alix, HSP70, and TSG101 was detected to confirm the identity of these structures as SEVs (Figure S6C), and the NTA results revealed that the particle sizes of SEVs from both M0Ms and M2Ms were approximately 100 nm (Figure S6D). To identify whether M2M-SEVs can be taken up by CFs, M2-SEVs were labeled with DiI, which emits strong red fluorescence. As highlighted by our fluorescence micrographs, these SEVs were taken up by CFs (Figure S6E). Collectively, our results indicated the successful isolation of SEVs from M0Ms and M2Ms and showed that these SEVs could be taken up by CFs.

### 4. Screening, verification, and cellular localization of circUbe3a in M2M-SEVs

**Figure 4:**
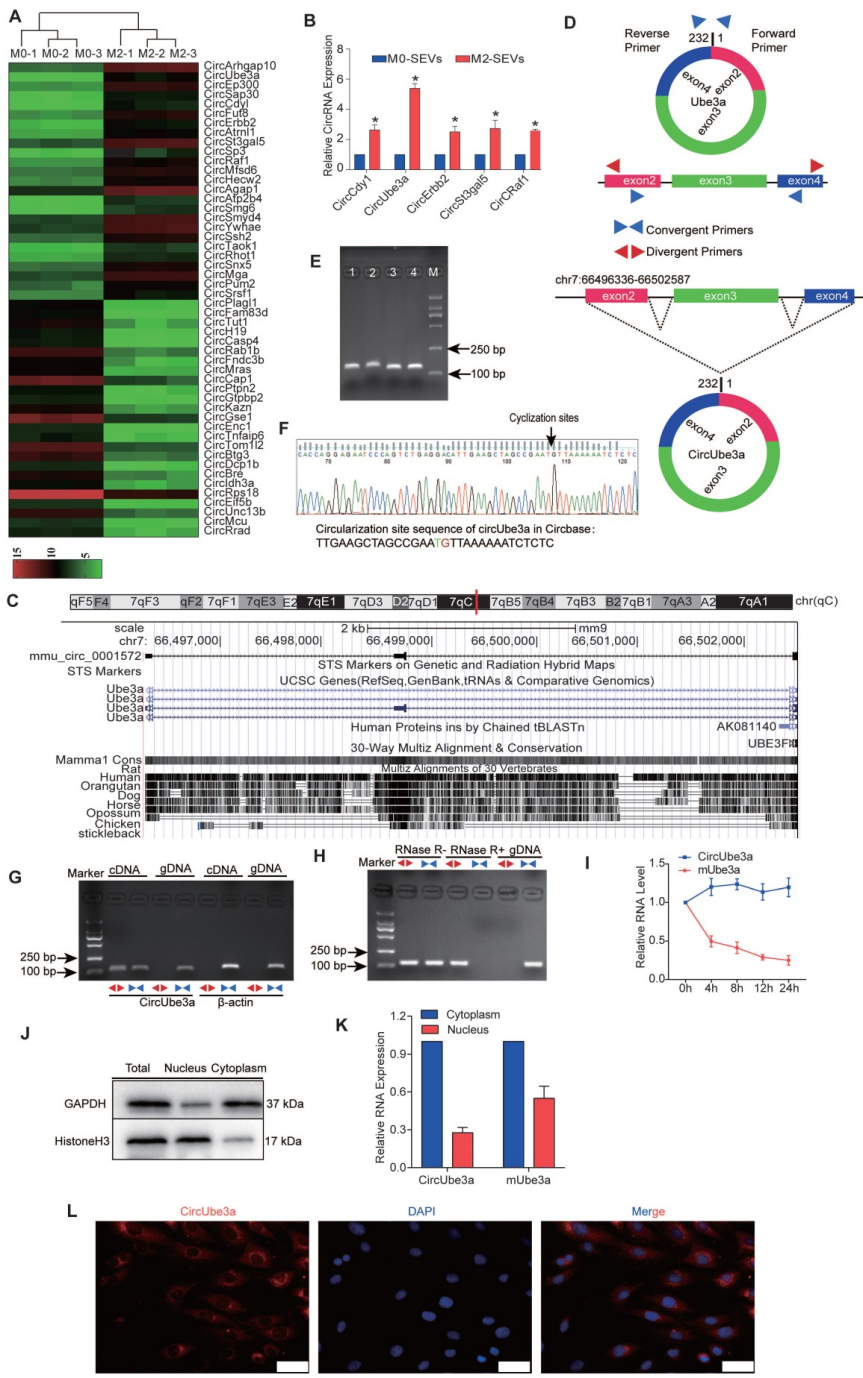
** Characteristics of circUbe3a carried in M2M-SEVs. A:** Differentially expressed circRNAs in M0M-SEVs and M2M-SEVs, as identified by microarray analysis. **B:** Differentially expressed circRNAs in M0M-SEVs and M2M-SEVs were further analyzed by RT-qPCR (*P < 0.05 vs M0M-SEVs)**. C:** CircUbe3a is located in the genome at chr7:66496336-66502587. A schematic diagram of circUbe3a formation is shown. **D:** Schematic diagram showing the positions of the divergent and convergent primers**. E:** Nucleic acid electrophoresis band. The molecular weight of the PCR product was approximately 100 bp. **F:** The sequence of circUbe3a in circBase (upper part) was consistent with the Sanger sequencing results (lower part). **G:** Nucleic acid electrophoresis revealed the PCR products amplified by the divergent and convergent primers. **H:** Nucleic acid electrophoresis revealed the PCR products before and after RNA enzyme treatment. **I:** The half-lives of circUbe3a and linear mUbe3a were measured after treatment with actinomycin D. **J:** RNAs and their encoded proteins in the cytoplasm and nucleus were extracted and isolated using a nucleocytoplasmic separation kit, and then Western blot analysis was performed to assess protein expression to evaluate the separation efficiency. **K:** qPCR was used to detect the nucleoplasmic expression of Ube3a linear mRNA and circRNA. **L:** RNA-FISH with Cy3-labeled probes was conducted to detect circUbe3a expression in CFs. Nuclei were stained with DAPI. Scale bar = 20 μm.

SEVs are endocytic membrane-derived vesicles that mediate cell-to-cell interactions and the delivery of proteins and RNAs 22. The role of M2M-SEVs in CFs and AMI model mice prompted us to determine the molecular mechanisms underlying these processes. Hence, microarray analysis was applied to identify the differentially expressed circRNAs in M0M- and M2M-SEVs, and a heatmap was then generated (Figure 4A). The levels of circCdyI, circUbea3a, circErbb2, circSt3gal5, and circRaf1 were elevated in M2M-SEVs, as further confirmed by RT-qPCR (Figure 4B). Among these circRNAs, circUbea3a showed the most significant enhancement and was therefore selected as the target molecule for subsequent experiments.

CircUbe3a, identified in the circBase database as mmu_Circ_0001572 (http://www.circbase.org/cgi-bin/singlerecord.cgiid=mmu_circ_0001572), is derived from the ubiquitin protein ligase E3A (Ube3a) gene and is genomically located at chr7:66496336-66502587. CircUbe3a is the 2nd circRNA formed by the backsplicing of exon 4, which was determined by combining circBase search data with CircPrimer software to draw a schematic diagram showing the process of circUbe3a formation by backsplicing (Figure 4C).

Next, we analyzed the circRNA expression patterns in polarized macrophages to determine the change in circUbe3a expression that could induce a functional change in CFs. We first verified the ring structure and its stability characteristics. Mmu-circUbe3a (ID: mmu_circ_0001572), located at chr7:66496336-66502587, is 6252 bp long and derived from the gene encoding Ube3a (Figure 4D). Nucleic acid electrophoresis revealed the PCR amplification products a single band corresponding to the correct molecular weight (Figure 4E). Gene sequencing was performed on the product to identify the splice site sequence in the circRNA and verify its ring-forming characteristics (Figure 4F). In agarose gel electrophoresis, the specific circRNA band appeared only in the cDNA sample amplified with divergent primers and not in the sample amplified with convergent primers (Figure 4G), suggesting that circUbe3a originated from the transcript but was not in the genome. Furthermore, we conducted quantitative analysis of the circRNA before and after RNase treatment, and the results were consistent with the agarose gel electrophoresis findings (Figure 4H). Actinomycin D was used to inhibit cell transcription, and the half-life of circUbe3a was significantly longer than that of linear mUbe3a (Figure 4I), suggesting that the characteristics of circUbe3a were consistent with the basic characteristics of a stable circRNA and that circUbe3a was not easily degraded by RNases.

To determine the cellular localization of circUbe3a in M2Ms, qPCR was used to detect the expression of Ube3a linear mRNA and circRNA in the cytoplasm. The results showed no significant differences in the expression of the linear mRNA in the cytoplasm, while circRNA was localized primarily in the cytoplasm (Figure 4J, 4K). Furthermore, FISH revealed circUbe3a localization in the cytoplasm, as evidenced by red fluorescence (Figure 4L). These results suggested that macrophages polarized into M2Ms could release circUbe3a-enriched SEVs and that circUbe3a was stably expressed and localized in the cytoplasm.

### 5. Role of circUbe3a in the regulation of CF migration and proliferation

To explore the effect of circUbe3a on the biological behavior of CFs, lentiviruses were used to stably overexpress circUbe3a or linear Ube3a, and the overexpression efficiency was verified by qPCR. The circUbe3a gene was successfully overexpressed without affecting the expression level of the corresponding linear gene transcript. By contrast, overexpression of the linear Ube3a gene transcript did not affect the expression level of circUbe3a (Figure S7A, S7B). The transwell results demonstrated that CF migration was significantly increased in the circUbe3a overexpression group (Figure S7C). The EdU staining results showed that circUbe3a overexpression significantly enhanced the proliferation capacity of CFs (Figure S7D). Analysis of the cell cycle distribution by FCM showed that the percentage of G0/G1-phase cells was significantly decreased but that of S + G2/M-phase cells was significantly increased in the LV-circUbe3a group compared with that of the control group (*P* < 0.05). However, compared with those in the control group, the percentages of G0/G1- and S + G2/M-phase cells in the LV-linear Ube3a group were not significantly altered (Figure S7E). Western blot analysis was used to determine the expression levels of proteins related to the phenotypic transformation of CFs in each group, revealing that collagen I, collagen III, and α-SMA levels were significantly increased in the circUbe3a overexpression group. However, linear Ube3a could not promote CF proliferation, migration, or phenotypic transformation (Figure S7F).

More importantly, we also performed a rescue experiment by suppressing circUbe3a to observe the effects on the proliferation, migration, and phenotypic transformation of CFs. Angiotensin II (Ang II) was used to establish a model of cell proliferation and phenotypic transformation. The promotive effect of Ang II on cell migration was weakened with si-circUbe3a transfection, while knockdown of linear Ube3a had no obvious effect (Figure S7G). The FCM results showed that si-circUbe3a effectively inhibited Ang II-induced cell cycle acceleration, while the knockdown of linear Ube3a did not inhibit Ang II-induced cell proliferation (Figure S7H). Moreover, the collagen I, collagen III, and α-SMA expression levels were significantly decreased in the si-circUbe3a group (Figure S7I).

### 6. Potential involvement of circUbe3a carried in M2M-SEVs in promoting the proliferation, migration, and phenotypic transformation of CFs

**Figure 5:**
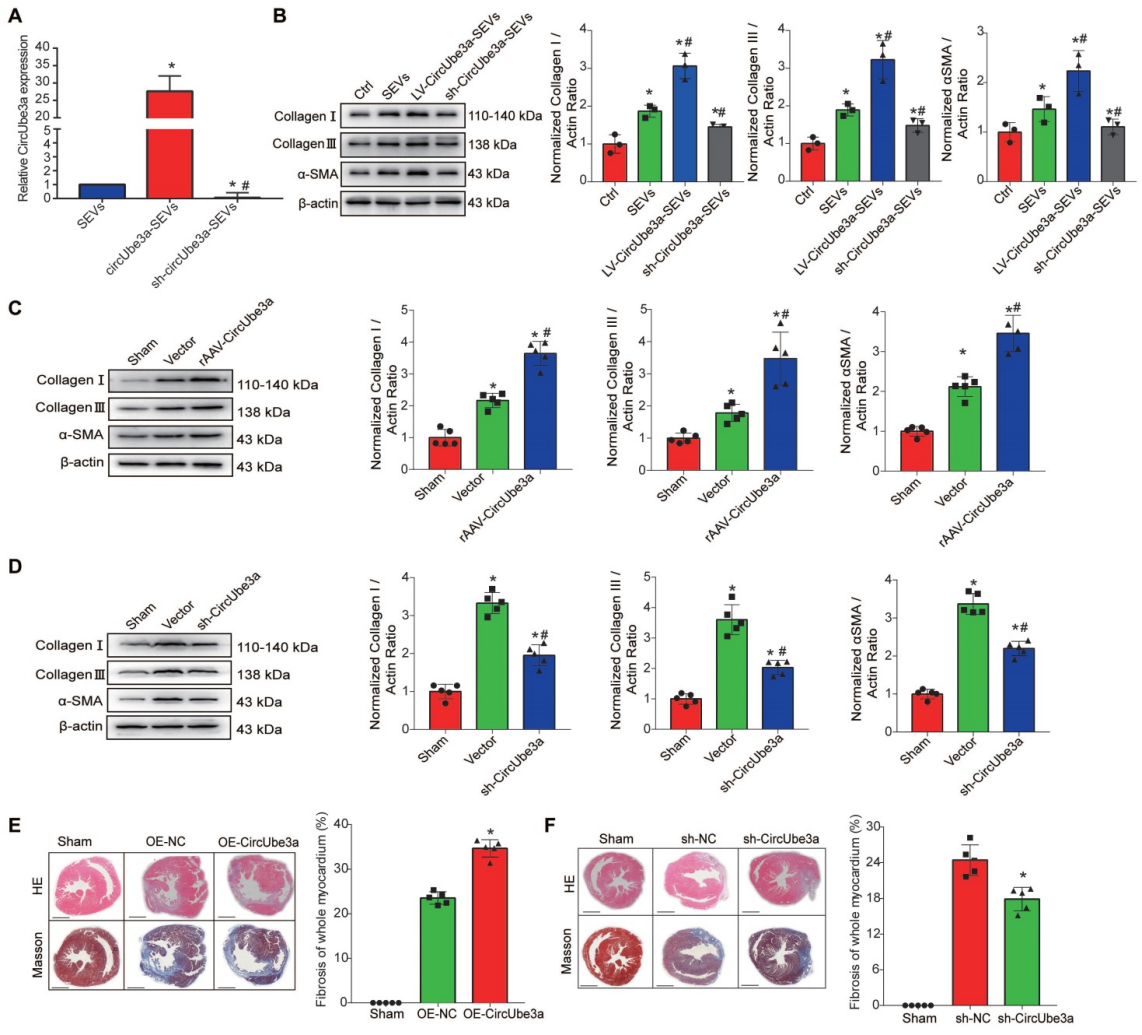
** circUbe3a promotes myocardial fibrosis after AMI. A:** Differentially expressed circUbe3a in circUbe3a-SEVs and sh-circUbe3a-SEVs was further assessed by RT-qPCR (*P < 0.05 compared with circUbe3a-SEVs). **B:** Representative Western blot results from three independent experiments and quantification of collagen I, collagen III, and α-SMA expression in recipient CFs treated with M2M-SEVs in the absence or presence of circUbe3a. (*P < 0.05 versus the Ctrl group; ^#^*P* < 0.05 versus the SEVs group; n = 3 per group). **C:** Representative Western blot results from five independent experiments and quantification of collagen I, collagen III, and α-SMA expression in cardiac muscle tissues treated with rAAV-circUbe3a or its NC. (*P < 0.05 versus the sham group; ^#^*P* < 0.05 versus the vector group; n = 5 per group).** D:** Representative Western blot results from five independent experiments and quantification of collagen I, collagen III, and α-SMA expression in cardiac muscle tissues treated with sh-circUbe3a or its NC (*P < 0.05 versus the sham group; ^#^*P* < 0.05 versus the vector group; n = 5 per group). **E:** Representative micrographs of left ventricular sections (H&E-stained and Masson's trichrome staining) and summary of the semiquantitative analysis of the Masson's trichrome-positive area. (*P < 0.05 versus the sham group; ^#^*P* < 0.05 versus the OE-NC group; n = 6 per group. Scale bars =200μm). **F:** Representative micrographs of left ventricular sections (H&E-stained and Masson's trichrome staining) and summary of the semiquantitative analysis of the Masson's trichrome-positive area (*P < 0.05 versus the sham group; ^#^*P* < 0.05 versus the sh-NC group; n = 6 per group. Scale bars =200μm).

To further clarify the transfer of circUbe3a by M2M-SEVs, we used viral vectors to obtain M2M-SEVs with stable up- or downregulation of circUbe3a expression (Figure 5A). We then cocultured those SEVs with CFs to observe the impact of SEV-derived circUbe3a on the phenotypic transition of CFs. Western blot analysis was used to detect the expression of phenotypic transformation-related proteins in CFs, revealing that treatment with SEVs overexpressing circUbe3a significantly promoted collagen I, collagen III, and α-SMA expression in fibroblasts (Figure 5B). Taken together, our results indicated that M2M-SEVs could mediate the proliferation, migration, and phenotypic transformation of fibroblasts through the transfer of circUbe3a.

### 7. CircUbe3a promotes cardiac fibrosis after AMI

To further verify the role of circUbe3a-mediated cardiac fibrosis after AMI *in vivo*, circUbe3a gain- and loss-of-function assays were used to evaluate fibrosis levels after AMI. Recombinant adeno-associated virus (rAAV) was used to overexpress or inhibit circUbe3a *in vivo*, and immunofluorescence and Western blotting were adopted to evaluate the efficiency of viral transduction. Two weeks after cardiac rAAV transduction, the positive expression of enhanced green fluorescent protein (eGFP) in heart tissue, as determined by immunofluorescence, was at least 70%, indicating that viral transduction was effective (Figure S8A). Western blot analysis was performed to detect eGFP expression in each group. Compared with that in the sham-operated group, the eGFP expression level in each subgroup transduced with rAAV-eGFP was statistically significantly increased (Figure S8B, S8C). These mice were then subjected to MI after viral transduction, and H&E staining, Masson's trichrome staining, and Western blot analysis were then used to estimate myocardial fibrosis (Figure 5C-5F). The infarcted areas in mice overexpressing circUbe3a were larger than those in control mice (*P*<0.05), and fibrosis and remodeling were more severe. Thus, inhibiting circUbe3a partially alleviated myocardial fibrosis after AMI.

### 8. CircUbe3a targets miR-138-5p

Investigating molecules bound to circRNAs can help better clarify the underlying mechanism of circRNAs. Exonic circRNAs in the cytoplasm can affect the expression levels of related miRNAs by competing with miRNAs with sponge-like adsorption activity and can also function as molecular scaffolds that play a regulatory role at the transcriptional level 32, 33. The studies described above showed that circUbe3a, which is highly expressed in M2M-SEVs, is derived from the exonic region and is localized primarily in the cytoplasm in CFs.

**Figure 6:**
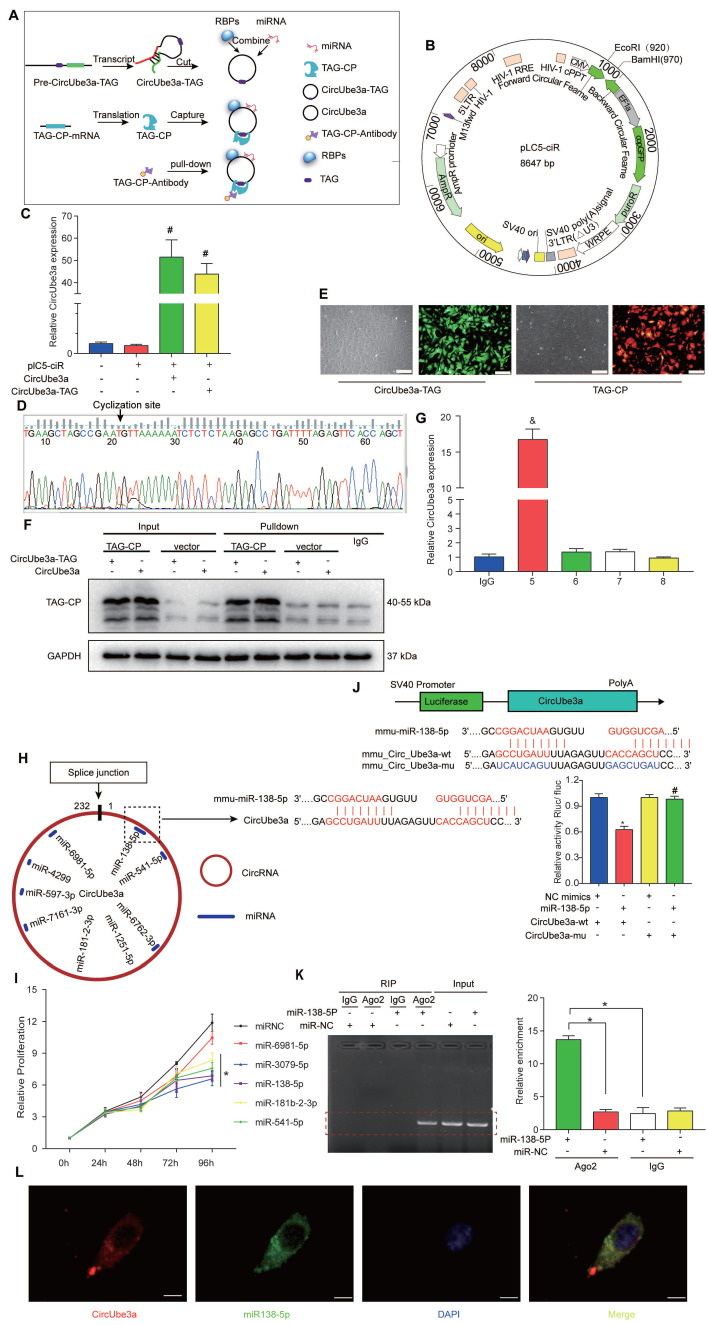
** circUbe3a acts as a miRNA sponge in CFs. A:** A schematic showing the procedure used for the circUbe3a pulldown assay. **B:** A schematic showing the pLC5-ciR vector. **C:** qPCR analysis of circUbe3a expression in CFs treated with circUbe3a or circUbe3a-TAG (^#^*P* < 0.05 versus the plC5-ciR group; n = 3 per group).** D:** The circUbe3a sequence, as determined by Sanger sequencing. **E:** Fluorescence micrograph of cells transfected with circUbe3a-TAG9 (green) or TAG-CP (red). Scale bars = 50 μm. **F:** Representative Western blot results from three independent experiments and quantification of TAG-CP protein expression before and after pulldown. **G:** qPCR analysis of circUbe3a expression in CFs before and after pulldown (IgG, circUbe3a-TAG+TAG-CP; 5, circUbe3a-TAG+TAG-CP; 6, circUbe3a+TAG-CP; 7, circUbe3aTAG+vector; 8, circUbe3a+vector; ^&^*P*<0.05 versus the IgG group). **H:** A schematic showing the putative binding sites of miR-138-5p in the circUbe3a transcript.** I:** CCK-8 assay of cells transfected with mimics of the principal miRNAs reported to play an important role in CF proliferation. Four miRNAs (miR-181b-2-3p, miR-541-5p, miR-138-5p, and miR-3070-5p) were verified to promote CF proliferation. However, only miR-138-5p was significantly upregulated (*P < 0.05 versus the miRNC group. n = 3 per group). **J:** The miR-138-5p binding sites in the circUbe3a sequence were mutated to construct the LUC-circUbe3a mutant vector. CFs were cotransfected with the LUC-circUbe3a mutant and different miRNAs. Luciferase activity was detected using a dual-luciferase assay at 48 h post transfection (*P < 0.05 versus the NC mimics+CircUbe3a-wt,group,^#^*P* < 0.05 versus the miR-138-5p+CircUbe3a-wt,group. n = 3 per group). **K:** The cytoplasmic and total cellular fractions were isolated from CFs and immunoprecipitated using an anti-Ago2 antibody or IgG. The amount of circUbe3a in the immunoprecipitate was detected by RT-qPCR (n = 3, *P < 0.05). **L:** FISH was conducted to detect circUbe3a (red) and miR-138-5p (green) expression in CFs. Scale bar = 10 μm.

CircRNAs often exert their biological effects by interacting with other molecules. In this experiment, a unique overexpression vector was designed to add an RNA tag to circRNAs. Gene chip technology and protein profiling technology can be used to capture circRNAs and their interacting molecules by establishing a highly specific and stable combination of RNA tags and capture proteins. These methods were applied to capture products to identify the target proteins or miRNAs that may interact with the circRNAs (Figure 6A). We first synthesized the sequences of circUbe3a and circUbe3a-TAG by whole-gene synthesis and then used EcoRI/BamHI double digestion to insert them into the lentiviral expression vector pLC5-ciR (Figure 6B). qPCR was then used to detect target gene expression. Compared with that in the pLC5-ciR vector control group, the expression level of circUbe3a was significantly increased in the circUbe3a and circUbe3a-TAG groups (Figure 6C). Moreover, the amplification product was subjected to Sanger sequencing, which revealed that the overexpression vector was accurately circularized (Figure 6D). The transfection efficiencies of the circUbe3a-TAG9 (green) and TAG-CP (red) expression vectors were determined by observation under a fluorescence microscope (Figure 6E). Western blot analysis was adopted to detect TAG-CP protein expression in samples before and after pulldown. The TAG-CP capture protein expression plasmid was added to each experimental group to express the capture protein, which was not expressed in the corresponding control group (Figure 6F). Additionally, circUbe3a was enriched by approximately 16-fold in the circRNA pulldown enrichment group (Group 5) compared with that in the IgG group (Figure 6G).

By screening the circRNA-miRNA databases miRanda and TargetScan, we determined that circUbe3a might bind 5 miRNAs: miR-138-5p, miR-6981-5p, miR-3079-5p, miR-181b-2-3p, and miR-541-5p (Figure 6H). Mimic overexpression vectors for these 5 miRNAs were constructed, and the CCK-8 assay showed that miR-138-5p, miR-3079-5p, miR-181b-2-3p, and miR-541-5p significantly inhibited the proliferation of CFs (Figure 6I). We selected miR-138-5p, which had the highest binding score for circUbe3a and inhibited the proliferation of CFs, as the target molecule for the subsequent experiment. The sequence of the complementary region in miR-138-5p and circUbe3a is shown in Figure 5I. Additionally, the dual-luciferase assay showed that circUbe3a and miR-138-5p had complementary binding sites (Figure 6J). RNA immunoprecipitation (RIP) was conducted using an anti-Ago2 antibody. The Ago2 protein pulled down circUbe3a, but the IgG control did not (Figure 6K), suggesting an interaction between miR-138-5p and circUbe3a. More importantly, RNA-FISH showed that circUbe3a and miR-138-5p colocalized in the cytoplasm in CFs (Figure 6L)*.*

### 9. CircUbe3a derived from M2M-SEVs mediates the proliferation, migration, and phenotypic transformation of CFs by targeting miR-138-5p

**Figure 7:**
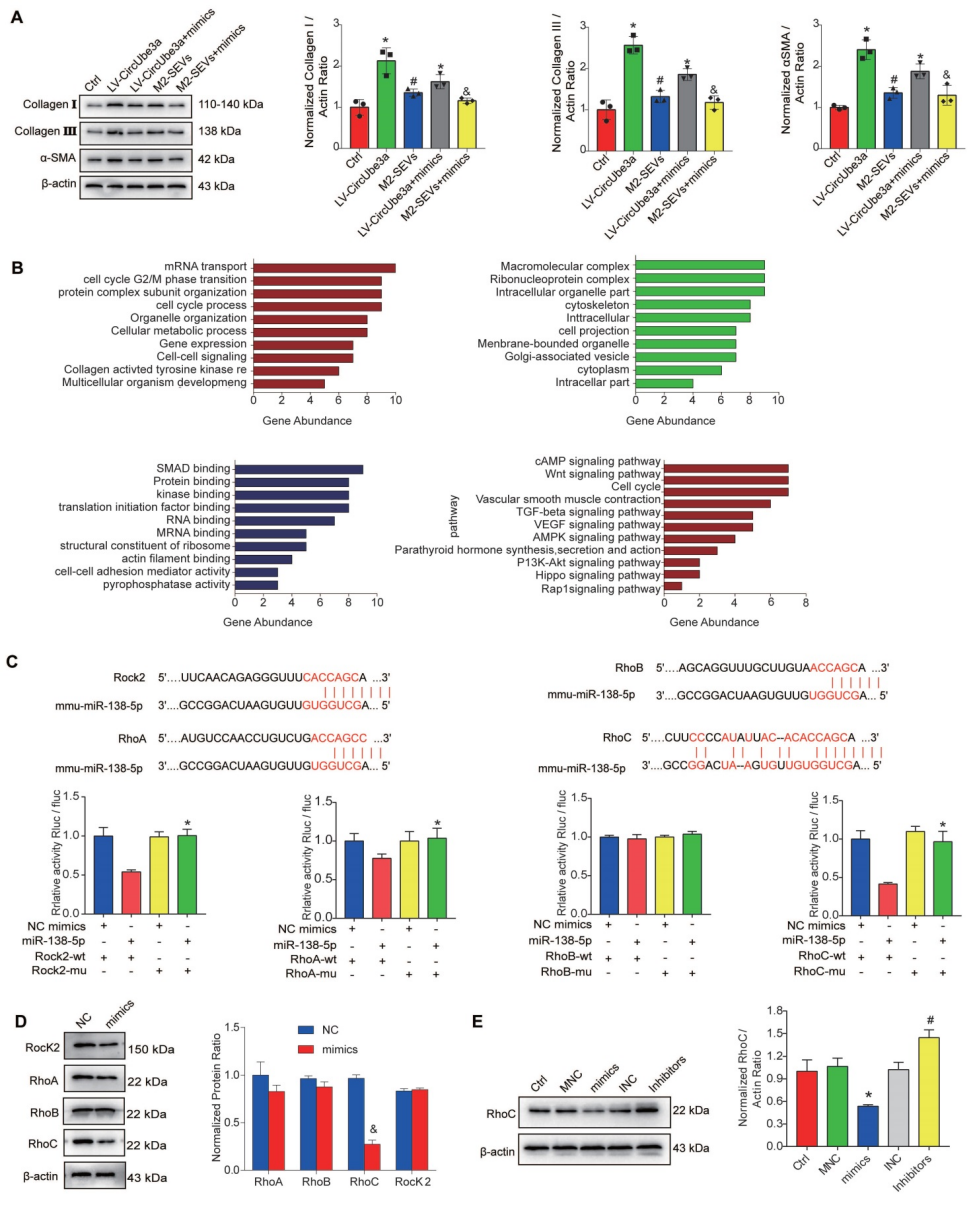
** Identification of the targeting relationship between miR-138-5p and RhoC**. **A:** Representative Western blot results from three independent experiments and quantification of collagen I, collagen III, and α-SMA expression in CFs treated with circUbe3a, circUbe3a+miR138-5p mimics, M2-SEVs, and M2-SEVs + miR-138-5p mimics (*P < 0.05 versus the Ctrl group; ^#^*P* < 0.05 versus the LV-circUbe3a group; ^&^*P* < 0.05 versus the M2-SEV group; n = 3 per group). **B:** Potential target genes downstream of miR-138-5p were subjected to GO term and KEGG pathway enrichment analyses; GO functional enrichment analysis included the biological process (BP), cell composition (CC), and molecular function (MF) categories. **C:** Sequence alignment of the miR‑138-5p and Rock2, RhoB, and RhoC 3'-UTR binding sites. Luciferase activity in the different groups after transfection. **D:** Representative Western blot results from three independent experiments and quantification of Rock2, RhoA, RhoB, and RhoC expression in CFs treated with miR-138-5p mimics (^&^*P* < 0.05 versus the NC group; n = 3 per group). **E:** Representative Western blot results from three independent experiments and quantification of RhoC expression in CFs treated with miR-138-5p mimics, miR-138-5p inhibitors, or the corresponding NCs (*P < 0.05 versus the Ctrl group; ^#^*P* < 0.05 versus the mimics group; n = 3 per group).

To evaluate the effects of miR-138-5p on the proliferation, migration, and phenotypic transformation of CFs, miR-138-5p mimics, inhibitors, and their corresponding NCs were transfected into CFs. The miR-138-5p mimics inhibited cell proliferation, migration and phenotypic transformation, while the miR-138-5p inhibitors demonstrated opposite effects (Figure S9A, S9B, S9C). Additionally, miR-138-5p reversed the effect of circUbe3a on the biological behavior of CFs. Next, the miR-138-5p mimics and circUbe3a overexpression vector were cotransfected into CFs. Western blot analysis showed that circUbe3a promoted the phenotypic transformation of CFs and that this effect was partially reversed by treatment with the miR-138-5p mimics; similarly, M2M-SEVs promoted the phenotypic transformation of CFs, while the miR-138-5p mimics partially reversed this effect (Figure 7A). The above results indicated that intervention with miR-138-5p reversed the effect of circUbe3a and M2-SEVs on the phenotypic transformation of CFs.

MiRNAs perform their functions primarily by influencing the expression levels of target genes. Exploration of the signaling pathways and biological functions of the miR-138-5p target genes indirectly reflect the possible biological effect of miR-138-5p. We performed Gene Ontology (GO) term and Kyoto Encyclopedia of Genes and Genomes (KEGG) signaling pathway enrichment analyses of the potential target genes downstream of miR-138-5p. As shown in Figure 7B, the miR-138-5p target genes were enriched mainly in the biological processes of mRNA transcription, the cell cycle, and collagen activation. Additionally, the miR-138-5p target genes were enriched primarily in the cellular components of macromolecular composition, vesicles, cytoskeleton, and cytoplasm and in the molecular functions of SMA binding, protein binding, and kinase binding. KEGG pathway enrichment analysis revealed that the target genes were mainly related to the cell cycle, TGF-β signaling pathway, and Wnt signaling pathway as well as to other signaling pathways closely associated with cell proliferation and fibrosis. To identify the downstream target genes of miR-138-5p, TargetScan, PicTar, and miRanda were used to predict the genes with potential binding sites. Using this information, along with software scoring data and data from previous reports, we finally selected Rock2, RhoA, RhoB, and RhoC for subsequent analysis. Interestingly, the dual-luciferase reporter assay showed that Rock2, Rhoa, and RhoC, but not RhoB, reduced luciferase activity (Figure 7C). Consequently, we selected Rock2, RhoA, and RhoC for further verification at the protein level. Western blot analysis showed that, in CFs transfected with miR-138-5p mimics, the expression levels of Rock2, RhoA, and RhoC were decreased and RhoC expression was decreased even more significantly (Figure 7D). These results indicate that RhoC may be the target gene of miR-138-5p.

The miR-138-5p mimics, inhibitors and their corresponding NCs were transfected into CFs to further evaluate RhoC expression. Western blot analysis showed that the RhoC expression was significantly reduced in the mimic group (*P* < 0.05) and significantly increased in the inhibitor group (Figure 7E). In summary, miR-138-5p might regulate the function of CFs by targeting RhoC.

### 10. CircUbe3a affects the biological behavior of CFs through the miR-138-5p/RhoC signaling axis

**Figure 8:**
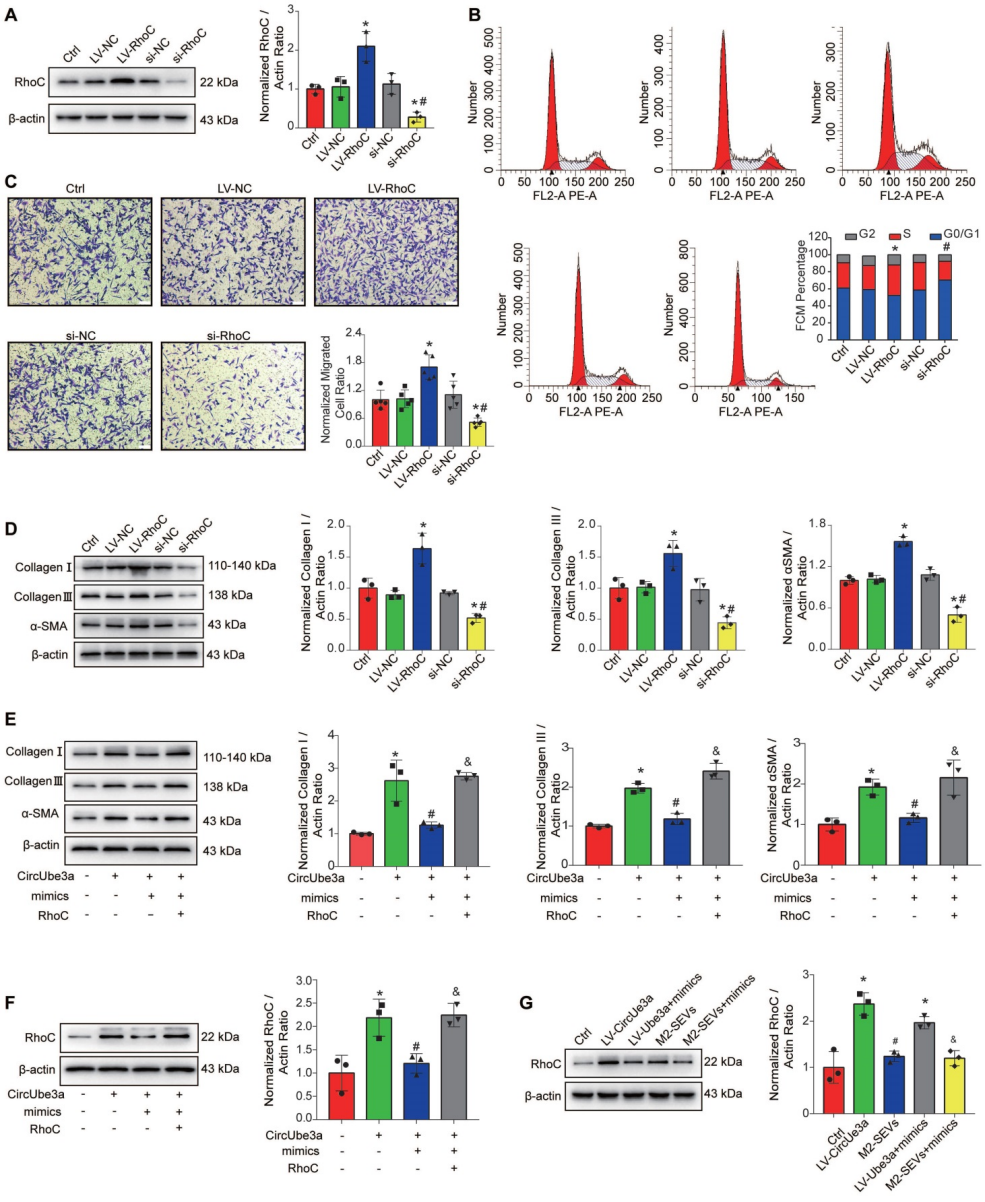
** The circUbe3a/miR-138-5p/RhoC axis regulates CF function *in vitro*. A:** Representative Western blot results from three independent experiments and quantification of RhoC expression in CFs treated with LV-RhoC or si-RhoC (*P < 0.05 versus the Ctrl group; ^#^*P* < 0.05 versus the LV-RhoC group; n = 3 per group).** B:** FCM indicated that RhoC was positively associated with cell cycle progression in CFs. (*P < 0.05 versus the Ctrl group; ^#^*P* < 0.05 versus the LV-RhoC group; n = 3 per group). **C:** A transwell assay was performed to assess CF migration after treatment with LV-RhoC or LV-siRNA-RhoC.(*P < 0.05 versus the Ctrl group; ^#^*P* < 0.05 versus the LV-RhoC group; n = 3 per group). **D:** Representative Western blot results from three independent experiments and quantification of collagen I, collagen III, and α-SMA expression in CFs treated with LV-RhoC or LV-siRNA-RhoC (*P < 0.05 versus the Ctrl group; ^#^*P* < 0.05 versus the LV-RhoC group; n = 3 per group)**. E:** Representative Western blot results from three independent experiments and quantification of collagen I, collagen III, and α-SMA expression in CFs treated with circUbe3a, circUbe3a+mimics, and circUbe3a +mimics+RhoC (*P < 0.05 versus the Ctrl group; ^#^*P* < 0.05 versus the circUbe3a group; ^&^*P* < 0.05 versus the circUbe3a + mimics group;n = 3 per group). **F:** Representative Western blot results from three independent experiments and quantification of RhoC expression in CFs treated with circUbe3a, circUbe3a+mimics, circUbe3a + mimics + RhoC (*P < 0.05 versus the Ctrl group; ^#^*P* < 0.05 versus the circUbe3a group;^ &^*P* < 0.05 versus the circUbe3a + mimics group; n = 3 per group).** G:** Representative Western blot results from three independent experiments and quantification of RhoC expression in CFs treated with circUbe3a, circUbe3a + mimics, M2-SEVs, and M2-SEVs + mimics (*P < 0.05 versus the Ctrl group; ^#^*P* < 0.05 versus the LV-circUbe3a group;^ &^*P* < 0.05 versus the M2-SEVs group; n = 3 per group).

RhoC promotes cell proliferation, migration, and phenotypic transformation 34. Overexpression of the RhoC gene in CFs influenced the biological functions of CFs (Figure 8A-8D). After inhibition of RhoC expression, the proliferation, migration, and phenotypic transformation abilities of CFs were weakened (Figure 8A-8D). To further confirm that circUbe3a can regulate RhoC through miR-138-5p to mediate the phenotypic transformation of CFs, we overexpressed miR-138-5p or RhoC in a rescue experiment (Figure 8E). The miR-138-5p mimics reversed the ability of circUbe3a to promote collagen I, collagen III, and α-SMA expression, while RhoC overexpression partially offset this effect of miR-138-5p. Additionally, circUbe3a promoted RhoC expression, which was also partially offset by miR-138-5p overexpression (Figure 8F). Interestingly, the regulatory effects of M2-SEVs RhoC were the same as those of circUbe3a (Figure 8G). Collectively, these findings indicated that circUbe3a in M2M-SEVs targeted miR-138-5p, thereby regulating the expression of RhoC and mediating the proliferation, migration, and myofibroblastic transformation of CFs.

## Discussion

In the present study, we demonstrated that M2M-SEVs are involved in mediating the proliferation, migration, and myofibroblastic transformation of CFs by targeting the miR-138-5p/RhoC axis via the transfer of circUbe3a to recipient cells. To our best knowledge, this study is the first to show that SEV-mediated intercellular communication between M2Ms and CFs may result in pathogenic ventricular remodeling after AMI.

After AMI, damaged and necrotic cardiomyocytes release numerous chemokines to recruit many immune inflammatory cells to the infarcted area, where they participate in repair and reconstruction after infarction 35. During the acute inflammatory reaction period, large numbers of monocytes infiltrate into the infarcted area and differentiate into M1Ms to mediate the inflammatory reaction by clearing phagocytic necrotic cells. As inflammation gradually subsides, polarization towards the M2 phenotype gradually increases, and M2Ms replace M1Ms as the dominant group of macrophages. M2Ms have anti-inflammatory effects and can stimulate the transformation of fibroblasts into myofibroblasts to maintain the stress structure of cardiac tissue after AMI 36, 37. However, long-term continuous stimulation of M2Ms can cause excessive fibrosis, which results in scarring and stiffening of cardiac tissue, affecting systolic function and leading to heart failure in severe cases 38. Therefore, a better understanding of the mechanism by which M2Ms regulate fibroblast activation may provide a reference for identifying new targets for anti-ventricular remodeling therapy.

Our results are consistent with previous reports showing obvious phenotypic changes in macrophages one week after MI; at 7 days after MI, macrophages and fibroblasts appeared in the infarcted area. These findings provide the basis for possible communication between macrophages and fibroblasts. EVs are responsible for horizontal gene transfer between cells to mediate intercellular communication 39. In the present study, EVs or conditioned medium from M2Ms but not EV-depleted conditioned medium promoted the proliferation, migration, and myofibroblastic transformation of CFs. Our study provides evidence that the increased incidence of myocardial fibrosis is attributable to M2M-EVs. Among these EVs, SEVs with particle sizes between 100 and 200 nm showed the most significant regulatory effect. Recently, an increasing number of studies have shown that ncRNAs carried by EVs are an important medium for the biological effects of EVs 40. CircRNAs are crucial mediators of the beneficial effects of exosomes (i.e., EVs with a diameter of approximately 100 nm) and can provide sustained therapeutic effects 10. However, little is known about which circRNAs in EVs are associated with M2M-induced functional changes in CFs. In the present study, differentially expressed circRNAs in macrophage-derived SEVs were detected by circRNA gene chip and qPCR analyses. Among these circRNAs, only circUbe3a exhibited significantly elevated levels in M2M-SEVs compared with those in M0M-SEVs.

The covalently closed loop structure is an important characteristic of circRNAs that makes them considerably different from other ncRNAs 41. Therefore, the circular structure of circUbe3a must be verified. Our study showed that amplification of cDNA, but not gDNA, with the divergent primer resulted in bright bands, suggesting that circUbe3a has a circular structure. We further analyzed the amplification product by Sanger sequencing to confirm that it contained the specific backsplicing sites. CircRNAs are resistant to RNase R digestion because of their closed covalent ring structure, are not easily degraded, and have a long half-life 25. CircUbe3a is mostly undigested, while its homologous mRNA is completely digested by RNase R. Similarly, the actinomycin D transcriptional inhibition assay showed that the half-life of circUbe3a was significantly longer than that of its homologous mRNA, suggesting that it has a stable covalent closed loop structure.

We speculate that circUbe3a, which is potentially effective as a connector, is abundant in M2M-SEVs. Moreover, circUbe3a promoted the proliferation, migration, and phenotypic transformation of CFs. To better simulate the physiological process by which circUbe3a is transferred into CFs through SEVs, we performed further experiments to alter the level of circUbe3a in M2-SEVs and observed the effects of M2-SEVs containing different amounts of circUbe3a on CFs. Introduction of circUbe3a siRNA significantly reduced the enhancing effects of M2M-SEVs on CF proliferation, a finding that was consistent with the effect of direct circUbe3a knockdown in CFs. Collectively, this compelling evidence suggested that we successfully linked M2-SEV-derived circRNAs with functional changes in CFs and that circUbe3a was the specific contributor in M2M-SEVs. However, the mechanism by which SEV circUbe3a affects the biological behavior of CFs is unclear, necessitating further experiments.

Exonic circRNAs, which are localized in the cytoplasm, contain binding sites for miRNAs and are involved in regulating numerous genes through their function as sponges 42. CircPrimer software suggested that circUbe3a is derived from exons 2-4 of the Ube3a gene located on chromosome 7 43. Cytoplasmic/nuclear separation and FISH revealed that circUbe3a is localized mainly in the cytoplasm, suggesting that circUbe3a may play a corresponding role by acting as a miRNA sponge. Next, we performed the circRNA pulldown assay and bioinformatic analysis and found that circUbe3a is complementary to miR-138-5p; the luciferase reporter assay confirmed this complementarity. Additionally, RNA-FISH revealed that circUbe3a is colocalized with miR-138-5p in the cytoplasm.

MiR-138-5p has been shown to inhibit cell proliferation, migration, and epithelial-mesenchymal transition in prostate tumors 44, lung adenocarcinomas 45, and breast tumors 46, among other tumors. Previous studies have demonstrated that the lncRNA KLF3-AS1 can sponge miR-138-5p, thereby exerting an antiapoptotic effect after MI 47. In the present study, miR-138-5p inhibited the proliferation, migration, and phenotypic transition of CFs, but these effects were restored by the miR-138-5p inhibitor. We also found that circUbe3a sponged miR-138-5p and then affected its activity but not its RNA expression level. MiR-138-5p overexpression partially reversed the effect of circUbe3a on the biological behavior of CFs. In summary, these results indicated that circUbe3a might play a role by sponging miR-138-5p. Subsequently, we verified that RhoC contained a binding site for miR-138-5p that could be targeted by miR-138-5p. More importantly, interfering with circUbe3a expression altered RhoC protein expression accordingly. However, intervention with circUbe3a expression did not alter miR-138-5p expression, indicating that circUbe3a could regulate RhoC expression. This effect was regulated at the posttranscriptional level by the sponging activity of miR-138-5p. RhoC plays an important role in the Rho signaling pathway and can function as a molecular switch in eukaryotic cells to control many signal transduction pathways 48, 49. Many studies have shown that RhoC affects cell cycle checkpoints by promoting cell proliferation, regulating actin cytoskeleton generation, and promoting cell migration 34, 50. In our study, RhoC overexpression promoted the migration of CFs, increased the expression of the cytoskeletal protein α-SMA, and enhanced myofibroblastic transformation of CFs. We speculate that RhoC may affect the acceleration of skeletal protein production, thereby increasing cell migration and myofibroblastic transformation. Surprisingly, the alteration of circUbe3a levels affected the expression of the miR-138-5p target protein RhoC, indicating that circUbe3a increases RhoC expression by inhibiting the activity of miR-138-5p. The present study demonstrated that circUbe3a transferred by M2-SEVs promotes the proliferation, migration, and myofibroblastic transformation of CFs through the miR-138-5p/RhoC axis.

In conclusion, the current study provided evidence that M2Ms may mediate cardiac fibrosis after MI by releasing SEVs containing circUbe3a and that circUbe3a regulates RhoC expression, which is reversed by miR-138-5p, ultimately indicating that circUbe3a plays a role in promoting the proliferation, migration, and myofibroblastic transformation of CFs through the circUbe3a/miR-138-5p/RhoC axis. However, circRNAs other than circUbe3a may also be carried by M2M-SEVs. Furthermore, we focused only on miR-138-5p and the downstream RhoC target protein, and some canonical fibrotic signaling pathways, such as the TGFβ/Smad pathway, were not examined in the current study. In our future studies, we will consider additional potential molecular mechanisms involved in M2M-SEV-mediated ventricular remodeling. The current findings provide new insights into the clinical use of SEVs in AMI treatment, and we anticipate that further studies will be conducted to validate our findings and develop additional therapeutic options.

## Supplementary Material

Supplementary figures and tables.

## Figures and Tables

**Table 1:** Sequences of PCR primers.

circUbe3a	F: 5'-ACCCTGATGTCACCGAATGG-3'
	R: 5'-TAGCTGCTAACTTGATCTGAACGT-3'
miR-138-5p	F: 5'-GGCCGGACTAAGTGTTGT-3'
	R: 5'-GCAGGGTCCGAGGTATTC-3'
U6	F: 5'-CTCGCTTCGGCAGCACATATACT-3'
	R: 5'-ACGCTTCACGAATTTGCGTGTC-3'
β-actin	F: 5'-TGAGCTGCGTTTTACACCCT-3'
	R: 5'-GTTTGCTCCAACCAACTGCT-3'
