# Supplementary material for: CircUbe3a from M2 macrophage-derived small extracellular vesicles mediates myocardial fibrosis after acute myocardial infarction
Source: Theranostics. 2021 Apr 15;11(13):6315–33. doi: 10.7150/thno.52843 (PMC8120198; doi:10.7150/thno.52843)
Supplement: Supplementary file 1 — Supplementary figures and tables. [file thnov11p6315s1.pdf]

CircUbe3a from M2 macrophage-derived small extracellular vesicles mediates myocardial fibrosis after acute myocardial infarction

Yan Wang[1&], Chaofu Li[1&], Ranzun Zhao[1&], Zhimei Qiu[1], Changyin Shen[2], Zhenglong Wang[1], Weiwei Liu[1], Wei Zhang[1], Junbo Ge[3] and Bei Shi[1*]

[1]Department of Cardiology, Affiliated Hospital of Zunyi Medical University, Zunyi 563000, China

[2]Department of Cardiology, The Second Affiliated Hospital of Zunyi Medical University, Zunyi 563000, China

[3]Department of Cardiology, Shanghai Institute of Cardiovascular Diseases, Zhongshan Hospital, Fudan University, Shanghai 200032, China

[&]Those authors contributed equally to this work.

Correspondence should be addressed to Bei Shi; shib@zmu.edu.cn

Supplementary tables and figures

Figure S1. EVs injected via the intramyocardial and tail vein routes are internalized by cardiomyocytes.

Figure S2. Echocardiography was performed to evaluate the effects of different EV injection methods on cardiac function in mice.

Figure S3. Morphological characteristics and identification of various types of macrophages.

Figure S4. Effects of EVs of different sizes on the proliferation of fibroblasts.

Figure S5. Comparison of the effects of M0M-, M1M-, and M2M-SEVs on the proliferation and migration of CFs.

Figure S6. Morphological characteristics and phenotypic identification of M0M- and M2M-SEVs.

Figure S7. CircUbe3a promotes the proliferation, migration and myofibroblastic transformation of CFs.

Figure S8. Assessment of the cardiac-specific circUbe3a overexpression and inhibition efficiencies via rAAV transduction.

Figure S9. miR-138-5p regulates the proliferation, migration and myofibroblastic transformation of CFs.

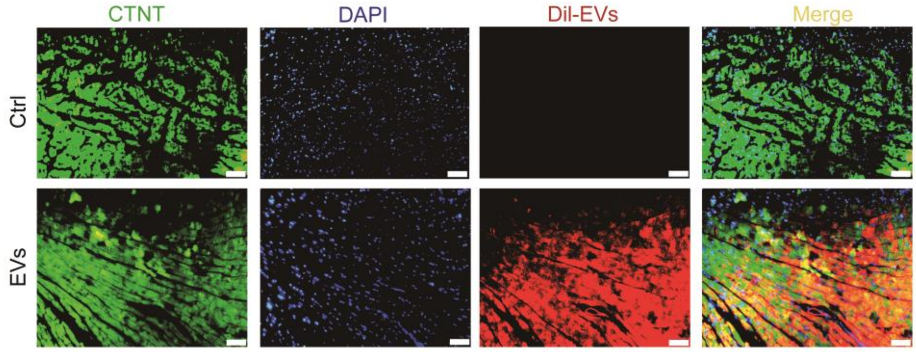

Figure S1. EVs injected via the intramyocardial and tail vein routes are internalized by cardiomyocytes (CMs). DiI-labeled M2M-EVs were injected into mice via the tail vein for 72 h. The relative fluorescence intensity (red) was quantified to identify the uptake of EVs by CMs. Scale bar=50 μm (n=5).

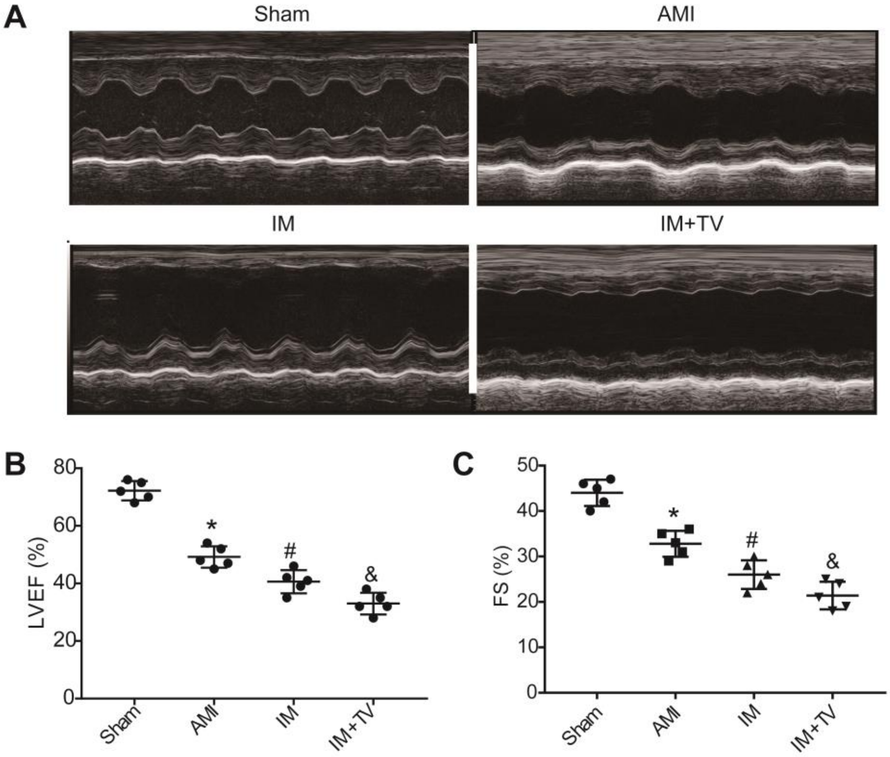

Figure S2. Echocardiography was performed to evaluate the effects of different EV injection methods on cardiac function in mice. A: Representative echocardiographic images. **B and C:** The LVEF (%) and FS values in the indicated groups were calculated (*P<0.05 versus the sham group; $^{\#}P$<0.05 versus the AMI group; $^{\&}P$<0.05 versus the IM group; n=5 per group).

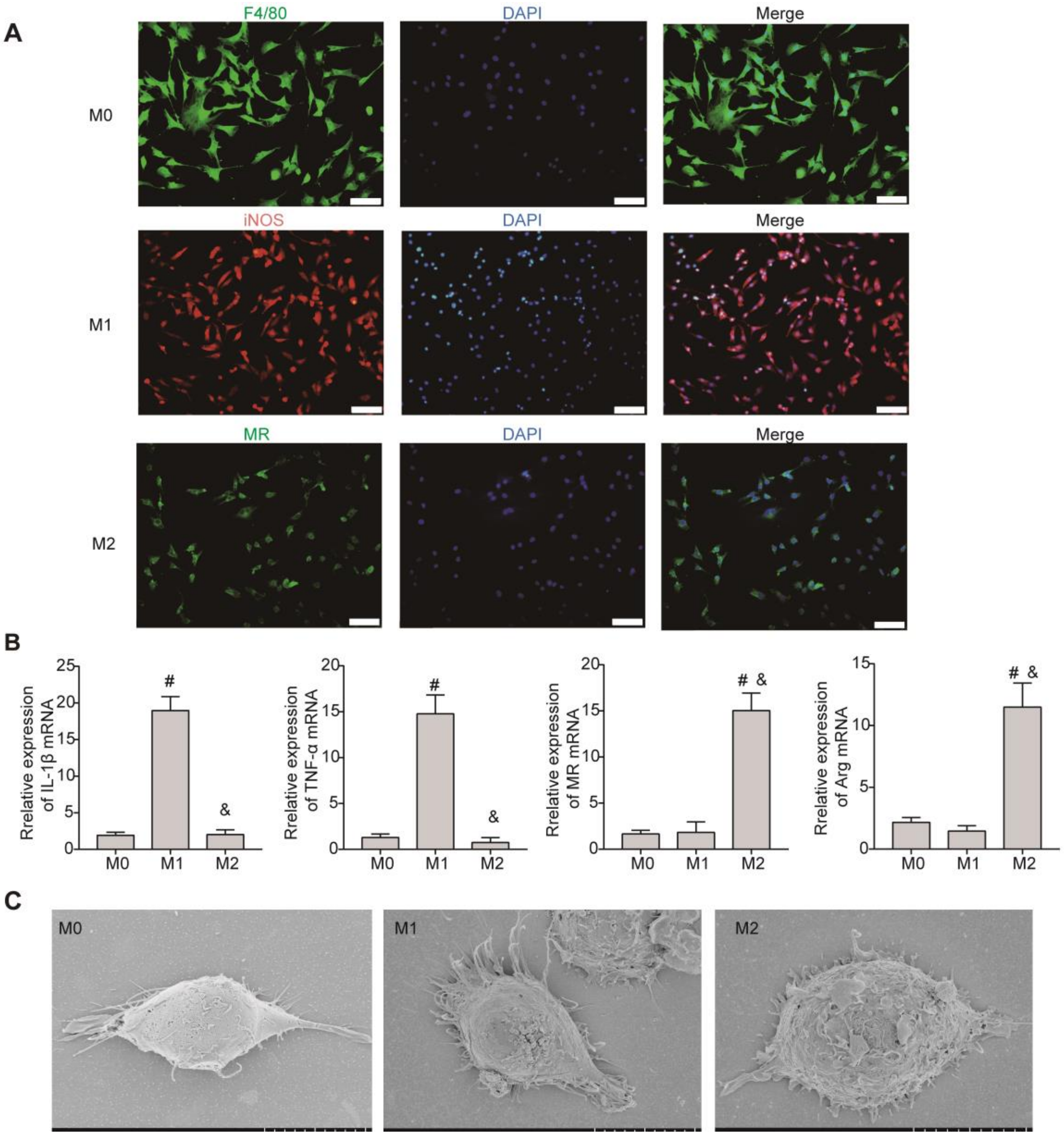

Figure S3. Morphological characteristics and identification of various macrophage types. A: Representative immunofluorescence images of surface markers on various macrophage types. Scale bar=50 μm. **B:** qRT-PCR was used to detect the expression of specific M1- and M2-related genes in various macrophage types. **C.**The morphology of macrophages was visualized by scanning electron microscopy.

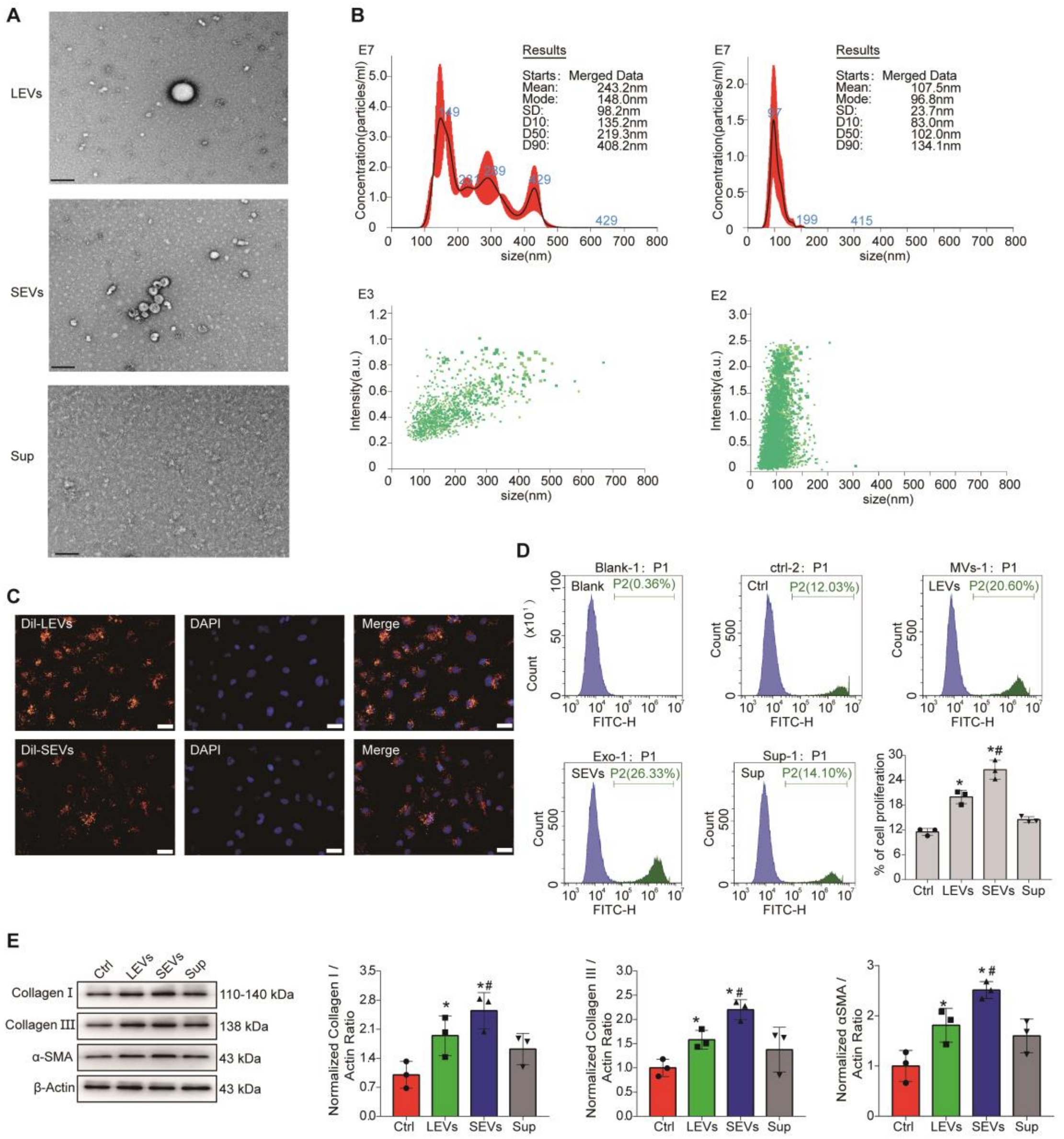

Figure S4. Effects of EVs of different sizes on the proliferation of fibroblasts. A: TEM demonstrated that the LEVs and SEVs secreted by M2Ms were cup-shaped vesicles. **B:** Size distributions of LEVs and SEVs isolated from M2M-conditioned medium, as determined by NTA. **C:** Uptake of LEVs and SEVs by CFs was assessed by immunofluorescence staining. Scale bar=50 μm. **D:** Effects of EVs of different sizes on CF proliferation, as determined by the EdU assay. **E:** Representative Western blot results from three independent experiments and quantification of collagen I, collagen III, and α-SMA expression in CFs treated with LEVs, SEVs and Sup (*$P<0.05$ versus the Ctrl group; $^{\#}P<0.05$ versus the LEVs group; n=3 per group).

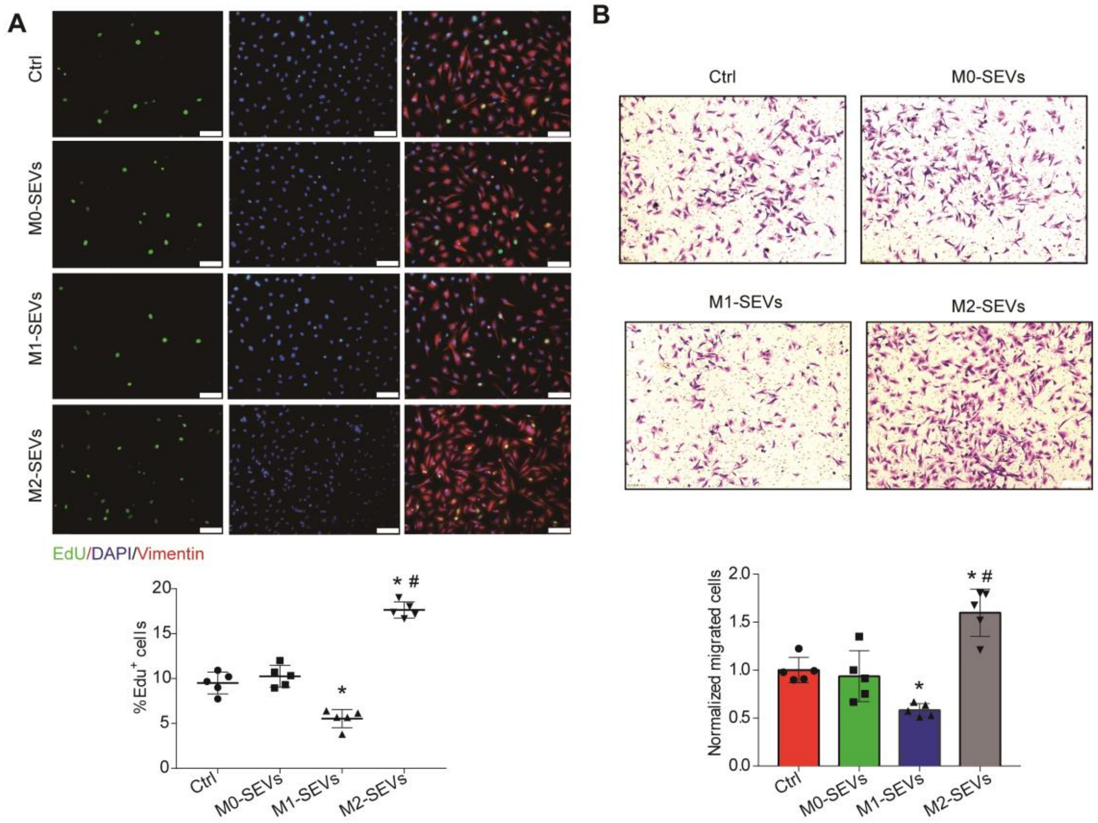

Figure S5. Comparison of the effects of M0M-, M1M-, and M2M-SEVs on the proliferation and migration of CFs. A: Representative immunofluorescence images showing the CF expression levels in different groups. The number of EdU-positive nuclei was used to assess cell proliferation. Scale bar=50 μm. **B:** The transwell assay was performed to evaluate the migration of CFs treated with M0-SEVs, M1-SEVs and M2-SEVs (*P<0.05 versus the Ctrl group; #P<0.05 versus the M0-SEVs group; n=3 per group).

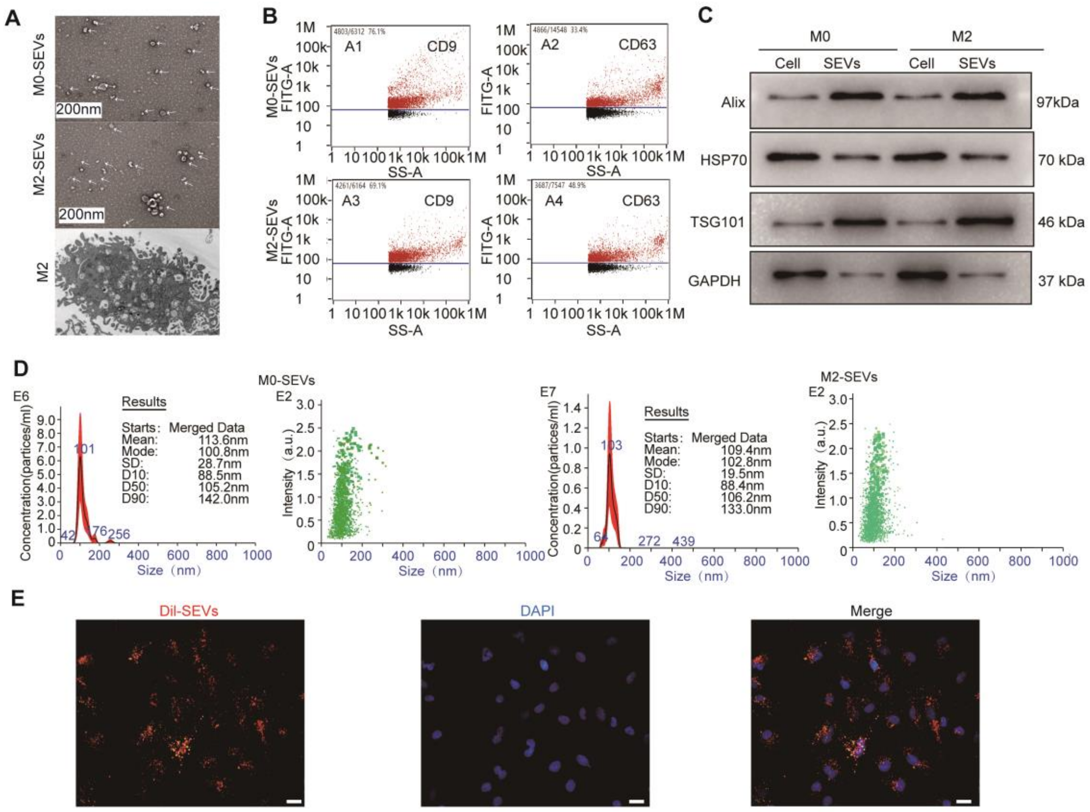

Figure S6. Morphological characteristics and phenotypic identification of M0M- and M2M-SEVs. A: TEM demonstrated that the SEVs secreted by M0Ms or M2Ms were cup-shaped vesicles. **B:** The markers CD63 and CD9 in M0M-SEVs and M2M-SEVs were detected by FCM. **C:** Alix, HSP70, and TSG101 protein expression was detected by Western blot analysis. **D:** Size distributions of SEVs isolated from M0M- or M2M-conditioned medium, as determined by NTA. **E:** The uptake of SEVs by CFs was measured by immunofluorescence staining. Scale bar=50 μm.

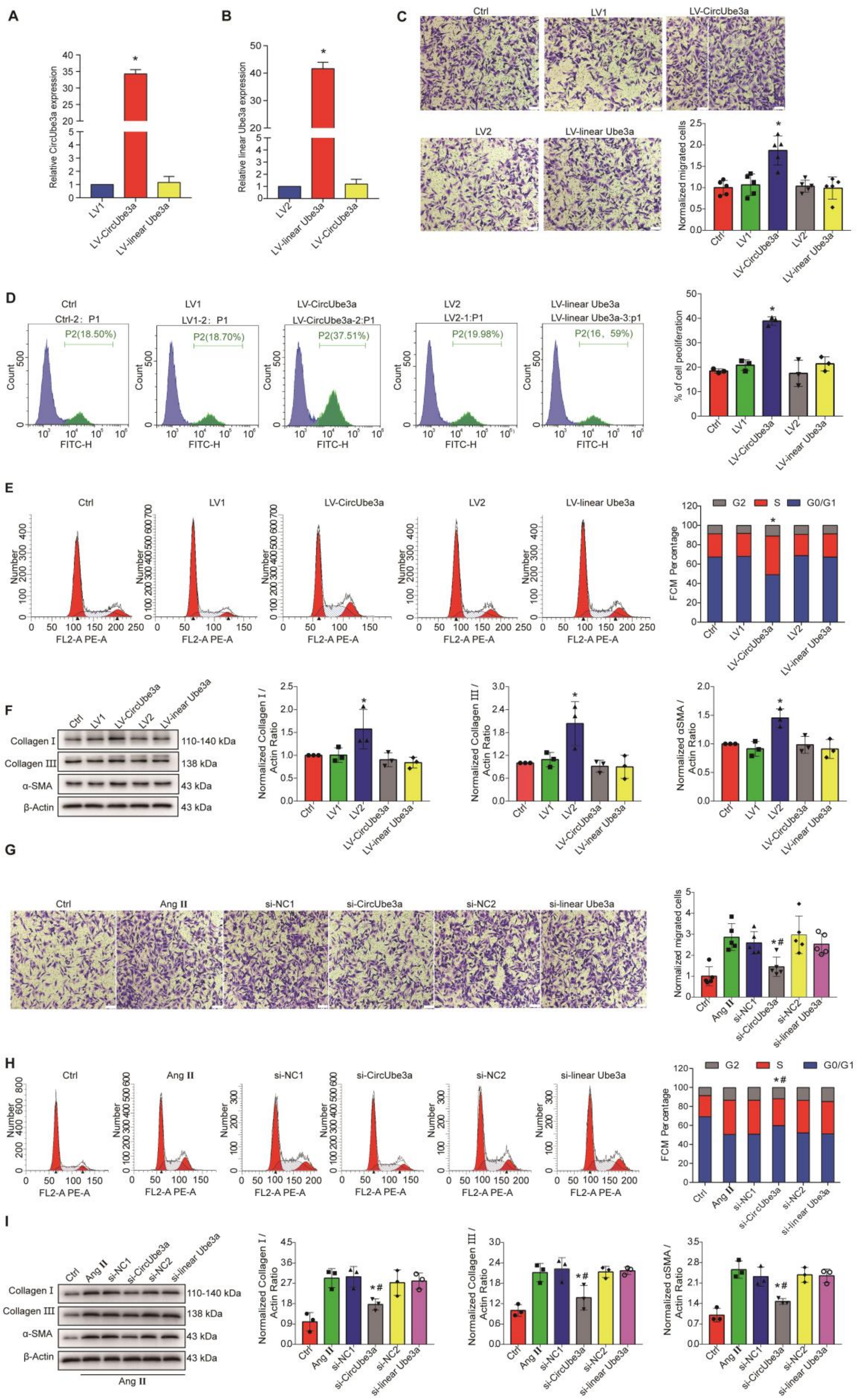
A
Relative CircUbe3a expression
LV1
LV-CircUbe3a
LV-linear Ube3a
B
Relative linear Ube3a expression
LV2
LV-linear Ube3a
LV-CircUbe3a
C
Ctrl
LV1
LV-CircUbe3a
LV2
LV-linear Ube3a
Normalized migrated cells
D
Ctrl-2：P1
P2(18.50%)
LV1-2：P1
P2(18.70%)
LV-CircUbe3a-2:P1
P2(37.51%)
LV2-1:P1
P2(19.98%)
LV-linear Ube3a-3:p1
P2(16，59%)
Count
FITC-H
% of cell peoliferation
E
Number
FL2-A PE-A
G2
S
G0/G1
FCM Percentage
F
Collagen I
Collagen III
α-SMA
β-Actin
110-140 kDa
138 kDa
43 kDa
Normalized Collagen I / Actin Ratio
Normalized Collagen III / Actin Ratio
Normalized αSMA / Actin Ratio
G
Ctrl
Ang II
si-NC1
si-CircUbe3a
si-NC2
si-linear Ube3a
Normalized migrated cells
H
Number
FL2-A PE-A
FCM Percentage
I
Collagen I
Collagen III
α-SMA
β-Actin
110-140 kDa
138 kDa
43 kDa
Ang II
Normalized Collagen I / Actin Ratio
Normalized Collagen III / Actin Ratio
Normalized αSMA / Actin Ratio

Figure S7. CircUbe3a promotes the proliferation, migration and myofibroblastic transformation of CFs. CFs were treated with LV-circUbe3a, LV-linear Ube3a, si-circUbe3a, si-linear Ube3a or their corresponding NCs to assess the effect of circUbe3a. **A and B:** qPCR analysis of circUbe3a and Ube3a mRNA expression in CFs ($^{*}P$<0.05 versus the LV1 group). **C:** Transwell assays were performed to assess CF migration after treatment with LV-circUbe3a or LV-linear Ube3a. **D:** The EdU assay was performed to evaluate CF proliferation after treatment with LV-circUbe3a or LV-linear Ube3a. **E:** FCM after the transduction of LV-circUbe3a or LV-linear Ube3a indicated that circUbe3a is positively associated with cell cycle progression in CFs. **F:** Western blot analysis of the protein expression of collagen I, collagen III, and α-SMA in cells treated with LV-circUbe3a or LV-linear Ube3a ($^{*}P$<0.05 versus the Ctrl group; n=3 per group). **G:** The transwell assay was performed to assess CF migration after treatment with si-circUbe3a or si-linear Ube3a. **H:** FCM after treatment with si-circUbe3a or si-linear Ube3a indicated that circUbe3a is positively associated with cell cycle progression in CFs. **I:** Western blot analysis of the protein expression of collagen I, collagen III, and α-SMA in cells treated with si-circUbe3a or si-linear Ube3a (*P<0.05 versus the Ctrl group; $^{\#}P$<0.05 versus the Ang II group; n=3 per group).

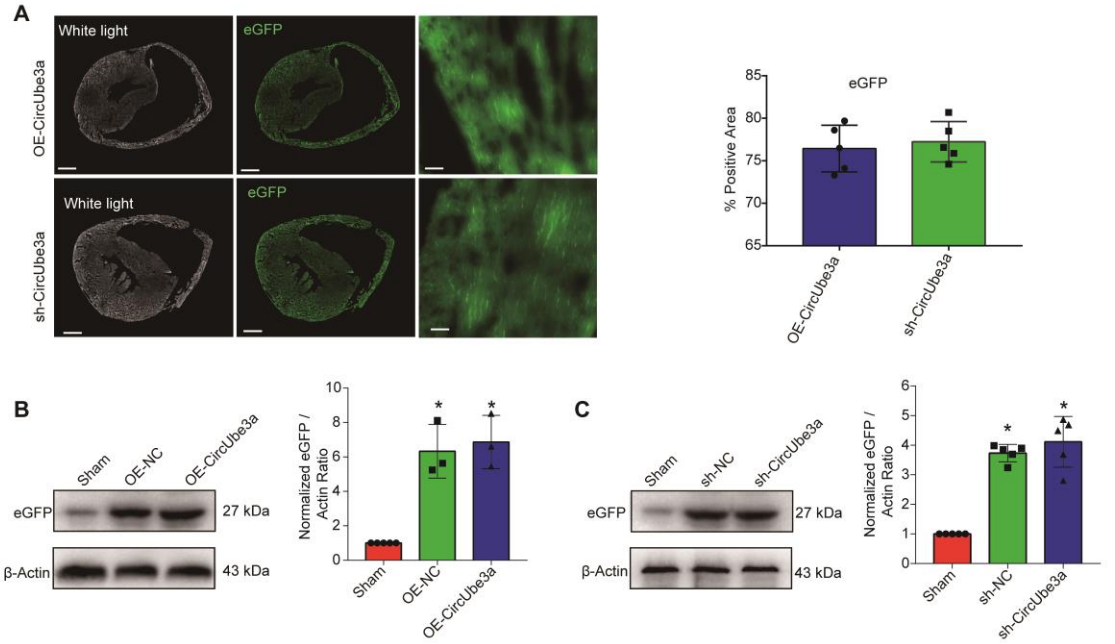

Figure S8. Assessment of the cardiac-specific circUbe3a overexpression and inhibition efficiencies via rAAV transduction. A: Immunofluorescence was used to detect the rAAV-mediated circUbe3a transduction efficiency. Scale bars=1000 μm. **B:**

Representative Western blot results from three independent experiments and quantification of eGFP expression in cardiac muscle tissues treated with rAAV-circUbe3a, rAAV-shcircUbe3a or its NC (*P<0.05 versus the sham group; n=6 per group).

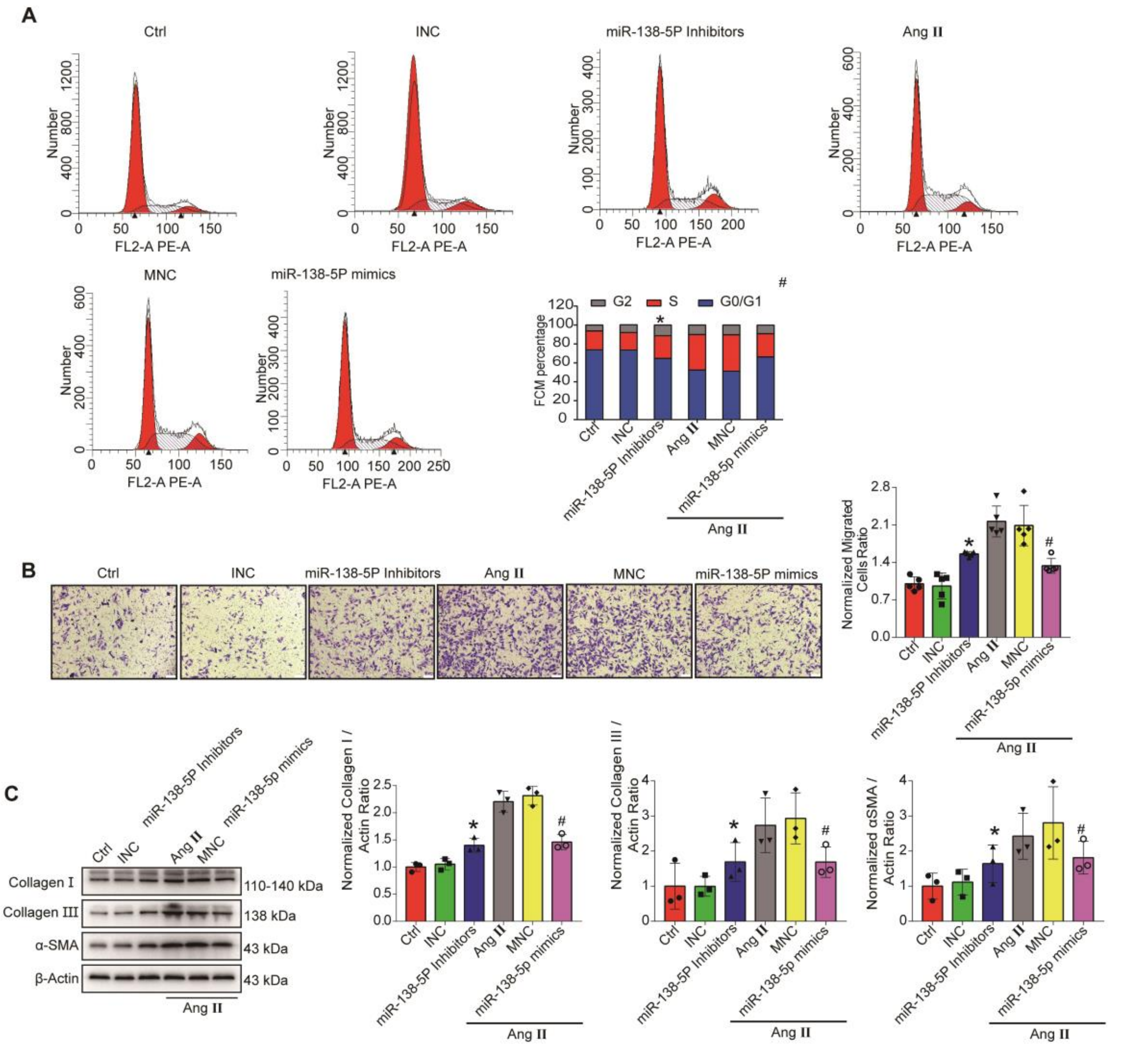

Figure S9. miR-138-5p regulates the proliferation, migration and myofibroblastic transformation of CFs. miR-138-5p mimics, inhibitors, and their corresponding NCs were transfected into CFs to assess the effect of miR-138-5p in CFs. **A:** FCM indicated that miR-138-5p is negatively associated with cell cycle progression in CFs. **B:** The transwell assay was performed to assess CF migration after treatment with miR-138-5p mimics or miR-138-5p inhibitors. **C:** Representative Western blot results from three independent experiments and quantification of collagen I, collagen III, and α-SMA expression in CFs treated with miR-138-5p mimics, miR-138-5p inhibitors or their

corresponding NCs (*$P<0.05$ versus the Ctrl group; #$P<0.05$ versus the miR-138-5p inhibitor group; n=3 per group).
